# Genomics discovery of giant fungal viruses from subsurface oceanic crustal fluids

**DOI:** 10.1038/s43705-022-00210-8

**Published:** 2023-02-03

**Authors:** Ananda S. Bhattacharjee, Frederik Schulz, Tanja Woyke, Beth N. Orcutt, Joaquín Martínez Martínez

**Affiliations:** 1 Bigelow Laboratory for Ocean Sciences, 60 Bigelow Drive, East Boothbay, ME USA; 2 US Department of Energy Joint Genome Institute, Berkeley, CA USA; 3 Present Address: Department of Environmental Sciences, University of California Riverside, Riverside, CA USA

**Keywords:** Virus-host interactions, Microbial ecology

## Abstract

The oceanic igneous crust is a vast reservoir for microbial life, dominated by diverse and active bacteria, archaea, and fungi. Archaeal and bacterial viruses were previously detected in oceanic crustal fluids at the Juan de Fuca Ridge (JdFR). Here we report the discovery of two eukaryotic Nucleocytoviricota genomes from the same crustal fluids by sorting and sequencing single virions. Both genomes have a tRNA^Tyr^ gene with an intron (20 bps) at the canonical position between nucleotide 37 and 38, a common feature in eukaryotic and archaeal tRNA genes with short introns (<100 bps), and fungal genes acquired through horizontal gene transfer (HGT) events. The dominance of *Ascomycota* fungi as the main eukaryotes in crustal fluids and the evidence for HGT point to these fungi as the putative hosts, making these the first putative fungi-Nucleocytoviricota specific association. Our study suggests active host-viral dynamics for the only eukaryotic group found in the subsurface oceanic crust and raises important questions about the impact of viral infection on the productivity and biogeochemical cycling in this ecosystem.

## Introduction

The oceanic igneous crust harbors the largest hydrologically active aquifer on Earth, and it is a site of dynamic exchange of fluids, chemical species, and microorganisms with the oceanic basin [1]. Phylogenetically and functionally diverse bacterial, archaeal, viral, and fungal taxa have been described in the oceanic basement [1]. Viruses are active participants in the ecology and evolution of microbial life [2]; yet, little is known about their diversity and activity in the marine crustal deep biosphere, with the main exception of a study revealing a diverse virus community in subsurface crustal fluids collected from the eastern flank of the Juan de Fuca Ridge (JdFR) [3]. These fluids were collected in 2011, 2013, and 2014 from borehole observatories installed in 2010 in Integrated Ocean Drilling Program (IODP) Holes U1362A (47°45.6628’N, 127°45.6720’W) and U1362B (47°45.4997’N 127°45.7312’W) [4]. The fluid samples were collected from 436 and 279 m below the seafloor, respectively [3]. Transmission electron microscopy and sequencing of microbial metagenomes obtained from tens to thousands of liters of fluid indicated that archaeon-infecting viruses and bacteriophages comprised the bulk of the viral community [3]. In addition, based on taxonomic affiliation, a small fraction of Nucleocytoviricota (Nucleocytoplasmic large DNA virus, NCLDV) genes were found within assembled scaffolds, but those genes were interpreted as belonging to archaeal viruses [3].

Nucleocytoviricota comprise a monophyletic group of double-stranded DNA viruses (70 kbp to 2.50 Mbp) [5] that infect a broad spectrum of unicellular and multicellular eukaryotes [6, 7] in many different habitats [8]. While their diversity is enormous [9, 10], they remain largely underexplored and poorly understood. Recent advances in metagenomics and targeted flow cytometry sorting and sequencing methods have improved the ability to capture and understand the genetic makeup of NCLDVs in environmental samples [9–18]. In this study, we sorted and sequenced the genome of single virus-like particles, including two NCLDVs, from a one-milliliter sample of crustal fluid collected in 2011 from the JdFR Hole U1362B. Here we report the taxonomic affiliations, and genomic characterization of those NCLDVS, and provide evidence of their ecology and interactions with putative hosts.

## Materials and methods

### Sample site and sampling

The oceanic basement fluid sample for this study was collected in July 2011 during cruise AT18-07 aboard the R/V *Atlantis* using ROV *Jason* II (cruise report available at: http://www.darkenergybiosphere.org/research/juandefuca.html). On dive J2-569, hydrothermal fluid samples were obtained from the Integrated Ocean Drilling Program (IODP) borehole U1362B (47°45.4997’N 127°45.7312’W) [19], located at an ocean depth of ~2650 m on the eastern flank of Juan de Fuca Ridge (JdFR) in the northeastern Pacific Ocean [20–22]. The sample was collected using a custom syringe sampler fired in the effluent of a free-flow chimney connected to the ball valve of the CORK (Circulation Obviation Retrofit Kit) well-head. The fluids originated from 359 m below the seafloor (~240 m of ocean sediments and 117 m into the basaltic basement) [3]. Upon recovery, a one-milliliter fluid aliquot was amended with 5% glycerol and 1 × TE (10 mM Tris-HCl, 1 mM EDTA, pH 8.0) (final concentrations) and stored at –80 °C until further processing for fluorescence-activated particle sorting.

### Virus single-amplified genome (vSAG) generation and sequencing

After thawing on ice, the sample was diluted 100-fold with sterile 0.2 µm-filtered 1×TE buffer and subsequently stained with SYBR Green I (Life Technologies, CA, USA), which has greater sensitivity for dsDNA, as described elsewhere [23]. The stained virus-like particles (VLPs), bacteria, and archaea were visualized by flow cytometry (Fig. S1A). Individual VLPs were sorted into one-half of a 384-well plate and bacteria/archaea cells in the other half. The plate layout included no-sort control wells (Fig. S1B). Sorting was performed at the Center for Aquatic Cytometry, Bigelow Laboratory for Ocean Sciences, with a BD Influx sorter following previously described methods [13].

Each well on the plate received 0.4 M KOH (final concentration) and was incubated at room temperature for 10 min to lyse the particles and render the gDNA available for amplification using Multiple Displacement Amplification (MDA) [24] (Fig. S1B). The MDA products from the whole plate were screened by PCR with primers for both bacterial and archaeal 16S rRNA genes as previously described [25] to confirm whether they derived from sorted cells or carry over cellular contamination (Fig. S1B). This work was carried out at the Single Cell Genomics Center, Bigelow Laboratory for Ocean Sciences. The 16S rRNA gene amplicons were used for phylogenetic analysis (Fig. S2).

Of the 27 putative vSAGs (i.e., successful MDA and no PCR-detectable 16S rRNA genes), eight were randomly selected for sequencing (vSAG1-8.JdFR). Nextera XT (Illumina, CA, USA) DNA libraries with a target size of 300 bp paired-end were prepared and sequenced on a MiSeq Benchtop sequencer (Illumina, CA, USA) at the Joint Genome Institute. Libraries from two aliquots of the same MDA amplicon were independently prepared and sequenced for vSAG1-8.JdFR.

### Sequence reads processing, assembly, and annotation

The Illumina raw reads (300 bps PE) were quality-trimmed with Trimmomatic v0.32 [26] using the following parameters: phred33 LEADING:0 TRAILING:5 SLIDINGWINDOW:4:15 MINLEN:36. Low complexity reads were removed using bbduk.sh from the BBTools suite [27] with a threshold of 0.5 (using entropy). Repairing of disordered quality filtered (QF) paired-end files was performed using repair.sh from BBTools suite. The QF reads for vSAGs were assembled using SPAdes v3.1.11 [28, 29] using the following parameters: --careful --sc --phred-offset 33 for the vSAGs. The scaffolds of at least 1000 bp were kept for further analyses. Six scaffolds (2.3–15.3Kbp) with 56–99% nucleotide level similarity present in all eight libraries were cautiously considered contamination and removed from further analysis. The contamination sequences are of bacterial and archaeal origin and are provided as supplementary data (https://github.com/asbhattacharjee/NCLDV-from-Juan-de-Fuca-Ridge-flank/blob/main/pre_annotation_contaimination_files/Pre_annotation_contamination%20sequence_list.xlsx).

The vSAGs’ coding sequences (CDS) were predicted using GeneMarkS [30] and their phyletic affiliations were determined through annotation using blastp (version 2.8.0+) [31] against the NCBI-NR database with e-value cut-off 10^–10^ (*February 2021*). The analysis was supplemented by blastp (version 2.8.0+) [31] analysis against NCBI Viral RefSeq (*February 2021*) and the reference viral database (RVDB) [32]. This analysis identified six of the vSAGs as bacteriophages. The remaining two, vSAG1.JdFR and vSAG8.JdFR, were identified as eukaryotic viruses. Only the latter two, vSAG1.JdFR and vSAG8.JdFR were retained for further analysis as part of this study. The vSAGs were further investigated for possible contaminations. We identified eleven scaffolds (1-17 kbp) in vSAG1.JdFR and vSAG8.JdFR libraries that were 100% identical at the nucleotide level to MarineAlpha5_Bin5 genes and had no viral genes. Those scaffolds were conservatively marked as putative contamination and removed from further analyses. All putative contamination sequences are made available as supplementary data (https://github.com/asbhattacharjee/NCLDV-from-Juan-de-Fuca-Ridge-flank/tree/main/post_annotation_contamination_files). The source of the putative contamination was not clear and was not further investigated in this study, although chemical analysis of the water sample and post-MDA screening with 16S rRNA suggested no contamination prior to sequencing. Post removal of putative contamination, a tBlastx [33] based alignment (nucleotide) of the vSAG1.JdFR and vSAG8.JdFR draft genomes was performed with the EasyFig [34] tool.

### vSAGs phylogenetic analysis

To infer the NCLDV species tree, hmmsearch [35] (version 3.1b2, hmmer.org) was used to identify five core NCVOGs NCLDV proteins [36, 37] using specific models (PMID: 28386012) for (i) DNA polymerase elongation subunit family B (NCVOG0038); (ii) D5-like helicase-primase (NCVOG0023); (iii) packaging ATPase (NCVOG0249); (iv) Poxvirus Late Transcription Factor VLTF3-like (NCVOG0262); and (v) DNA or RNA helicases of superfamily II (NCVOG0076). Proteins were aligned with mafft v7.294b [38], and positions with less than 10% of information were removed from the alignment with trimAl version1.4 [39]. Proteins were then concatenated, and a tree was built with IQ-Tree 1.6.6 [40] and FastTree [41] using the best-fit highest-scoring evolutionary model based on the ModelFinder [42] feature in IQ-Tree data: LG + F + R86. In addition to vSAG1.JdFR and vSAG8.JdFR, the tree included 117 public NCLDV genomes (https://github.com/asbhattacharjee/NCLDV-from-Juan-de-Fuca-Ridge-flank/tree/main/species_tree) which were pre-clustered at ANI 95% (alignment fraction >70%) with FastANI 1.0 [43].

We used blastp (version 2.8.0+) [31, 33] against the NCBI-NR database for the phylogenetic analysis of the vSAGs’ translational component eukaryotic initiation factor 4E (efl-4E), a common gene among Mesomimiviridae members. The ten top hits were extracted and aligned with mafft v7.294b [38] and the gaps in the alignments were removed with alignment with trimAl version1.4 [39]. The phylogenetic tree of the trimmed, concatenated proteins was constructed with PhyML [44], which includes automatic selection of the best-fit substitution model, LG + G + F (Fig. S4).

Orthofinder 1.03 [45] was used to infer clusters of orthologous genes (COGs) on a representative dataset of 215 NCLDV genomes for comparative analysis. The presence and absence of genes in COGs, genes shared between NCLDV lineages, vSAG1.JdFR and vSAG8.JdFR were computed to understand the evolutionary relatedness of novel JdFR giant viruses. A custom python script [18] that includes the packages matplotlib [46] and UpSet [47] was used to visualize the genes shared between (a) different NCLDVs; and (b) within vSAGs. The analysis files are provided as supplementary data (https://github.com/asbhattacharjee/NCLDV-from-Juan-de-Fuca-Ridge-flank/tree/main/orthogroups).

### tRNA prediction and phylogeny

The tRNA gene predictions and their structures for vSAG1.JdFR and vSAG8.JdFR and other mesomimiviruses (Table S1) were performed using tRNAscan-SE [48] (parameters: mixed tRNA model) and ARAGORN v1.2.38 [49] (parameters: Type tRNA and tmRNA; Allow introns, 0-3000 bases; linear sequence topology; both single and double-strand) with default search mode settings. The predicted NCLDV Tyrosine tRNAs were analyzed with blastn (version 2.8.0+) [31] against the NCBI-NR database to determine their phylogeny. The top hits (a total 37) were used for phylogenetic analysis. The tRNA sequences were aligned on MEGA X [50] with MUSCLE [51] and trimmed with trimmed with trimal version1.4 [39]. Phylogenetic position of the NCLDV tRNAs with introns at canonical positions was determined with a maximum likelihood tree, bootstrap value of 100.

### Horizontal gene transfer analysis

Top 100 blastn vs nr hits of the blastp [31, 33] against the NCBI-NR database were used as references for phylogenetic analysis of translation system proteins to identify potential horizontally transferred genes. The translation system proteins were aligned with mafft-linsi v7.294b [38] and positions with less than 10% of information were trimmed with trimal 1.4 [39] >90% gaps. The trees were then built with IQ-tree 1.6.6 [40] with the mixture model C40 + Rx (best fit model based on a model test [42]).

### Phyletic affiliations

The phyletic affiliations of the genes from Juan de Fuca Ridge metagenomes from the IODP boreholes U1362A and U1362B [3] published on Integrated Microbial Genomes and Microbiomes (IMG/M) [52, 53] were determined as described above for the vSAG1.JdFR and vSAG8.JdFR (Fig. S3A, B). The viral gene homolog family affiliations of borehole 1362B JdFR metagenome (IMG metagenome ID 3300002532) were categorized as NCLDVs, other dsDNA viruses and Unclassified dsDNA viruses. The genes considered for analysis were, (a) non duplicates, and (b) best blast hits of >30% identity match.

Genes from JdFR metagenomes affiliated to eukaryotes were sorted based on best blast hits, (a) 1362B borehole (IMG metagenome ID 3300002532), with >90% similarity, and (b) U1362A borehole (IMG metagenome IDs 3300002481) with, >60% but less than <90% similarity, as no hits had >90% similarity (Fig. S5A, B).

### Analysis of 18S rRNA, 28S rRNA, and internal transcribed spacer (ITS) sequences

The taxonomic classification of the 18S rRNA gene, 28S rRNA gene, and ITS sequences from the IODP borehole U1362B assembled metagenome [52, 53] was done by blastn search [31, 33] against the SILVA database [54] (Table S2).

## Results

**Fig. 1:**
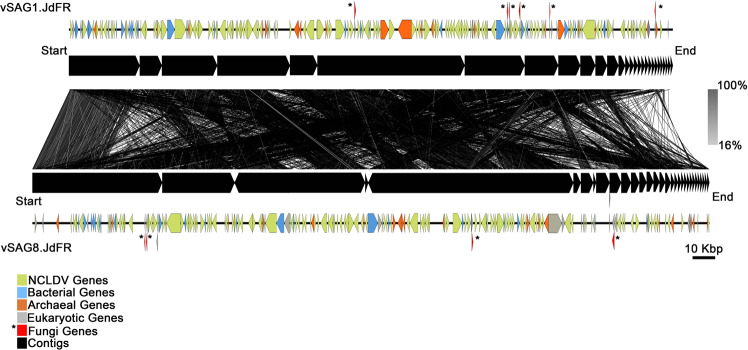
Nucleotide level alignment of vSAG1.JdFR and vSAG8.JdFR to each other. The vSAG1.JdFR (28 scaffolds) and vSAG8.JdFR (33 scaffolds). Black tick marks denote the breaks between the concatenated (using 10 Ns) scaffolds. The lines between the vSAG1.JdFR and vSAG8.JdFR genomes represent the nucleotide level alignment based on tBlastx analysis (alignment ranging from 16 to 100%). The predicted genes were assigned taxonomic affiliation based on Blastp analysis. Genes affiliated to bacteria (blue), archaea (orange), NCLDV (green), eukaryotes (gray), fungal (red), and scaffolds (black) for vSAG1.JdFR and vSAG8.JdFR on the genome.

Among the single-amplified genomes of particles sorted from the crustal fluid sample, we retrieved two related (average nucleotide identity (ANI) of 99.8%, alignment fraction of 70%) draft NCLDV genomes (vSAG1.JdFR and vSAG8.JdFR; Fig. 1, Tables S3 and S4). These NCLDV vSAGs were not represented in the metagenomes derived from samples collected at the same time from Holes U1362 A and U1362B [3]. However, our protein homology family affiliation analysis through the Integrated Microbial Genomes and Microbiomes (IMG/M) system [52] indicated that 3.31% of the unique viral genes and 25% of the total viral genes within the U1362B metagenome corresponded to NCLDVs (IMG ID 3300002532, Fig. S3).

### vSAG1.JdFR and vSAG8.JdFR belong to the mesomimiviridae family

**Fig. 2:**
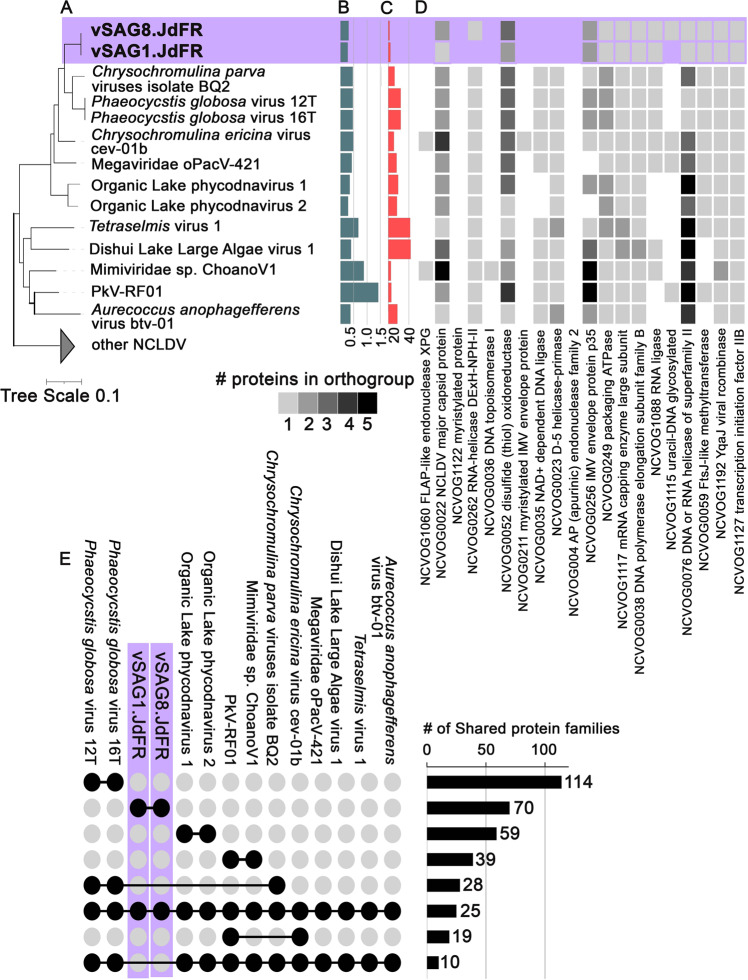
Phylogeny, genome characteristics, and genes identified in giant fungal viruses in oceanic crust. **A** Phylogenetic position (maximum likelihood tree) of Juan de Fuca Ridge viral single-amplified genomes (vSAG1.JdFR and vSAG8.JdFR) based on a concatenated alignment of five core nucleocytoplasmic virus orthologous genes (NCVOGs) (1. DNA polymerase elongation subunit family B (NCVOG0038); 2. D5-like helicase-primase (NCVOG0023); 3. packaging ATPase (NCVOG0249); 4. Poxvirus Late Transcription Factor VLTF3-like (NCVOG0262), 5. DNA or RNA helicases of superfamily II (NCVOG0076)). The scale bar represents substitutions per site; (**B**) genome assembly sizes (Mbp); (**C**) number of NCVOGs identified in each genome; (**D**) heatmap of protein copy number of genes mapped to the twenty ancestral NCVOGs. Presence/absence and protein copy number patterns of these genes compared to closest related viruses in the phylogenetic tree are indicative of genome completeness; (**E**) number of protein families shared between selected members of the mesomimiviruses. Each set of compared genomes is displayed as solid circles connected by horizontal solid lines; the number of shared protein families and total number of distinct protein families in each lineage are shown as bars.

Phylogenetic placement based on the concatenated alignment of five nearly universal NCLDV genes [55] showed vSAG1.JdFR and vSAG8.JdFR as sister lineages of *Phaeocystis globosa* viruses (PgVs) and *Chrysochromulina parva* viruses BQ2 (CpV BQ2) within the Mesomimiviridae family [56–58] (Fig. 2A). At the nucleotide level, vSAG1.JdFR and vSAG8.JdFR had ~65% and ~66% ANI with the PgVs and ~67 % and 69% ANI with CpV BQ2, respectively. Mapping the predicted genes from the relatively small vSAG1.JdFR and vSAG8.JdFR draft genomes (Fig. 2B) to the twenty established families of ancestral nucleocytoplasmic virus orthologous groups (NCVOGs [37]) showed that our vSAGs contain at least 11 and 13 ancestral NCVOGs, respectively (Fig. 2C, D; Table S5). The NCVOG presence/absence pattern for both vSAGs was most similar to other Mesomimiviridae viruses (Fig. 2D) and supported their evolutionary affinity. The vSAG1.JdFR and vSAG8.JdFR share 70 protein families (Fig. 2E) not identified in other Mesomimiviridae (as of *February 2021*), extending the Mesomimividae subfamily’s gene repertoire known so far (supplementary data: https://github.com/asbhattacharjee/NCLDV-from-Juan-de-Fuca-Ridge-flank). On the other hand, the vSAGs lack 10 protein families present in previously known Mesomimiviridae members included in our study (Fig. 2E).

### vSAG1.JdFR and vSAG8.JdFR contain putative bacterial, archaeal, and eukaryotic genes

The partial vSAG1.JdFR and vSAG8.JdFR genomes harbor genes from bacteria (at least 32 and 43, respectively), archaea (at least 20 and 25, for vSAG1.JdFR and vSAG8.JdFR, respectively), and eukaryotes (at least 17 and 20, respectively), based on annotation using the NCBI-NR database (*as of February 2021*) (Fig. 1, and Tables S6, S7, and S8). However, as previously shown [9, 59], potential misclassifications of genes in databases, such as NCBI-NR, cannot be completely ruled out, particularly in the case of some of those previously classified as archaeal genes [9].

The bacterial and archaeal genes (Fig. 1, blue and orange, respectively) found on scaffolds with NCLDV genes are clustered in islands across the genomes. Taxonomic identification of the genes predicted as archaeal in vSAG1.JdFR and vSAG8.JdFR showed that twenty were affiliated to Euryarchaeota also present within crustal fluids at this site, but not bottom seawater (Table S6, [22]).

**Fig. 3:**
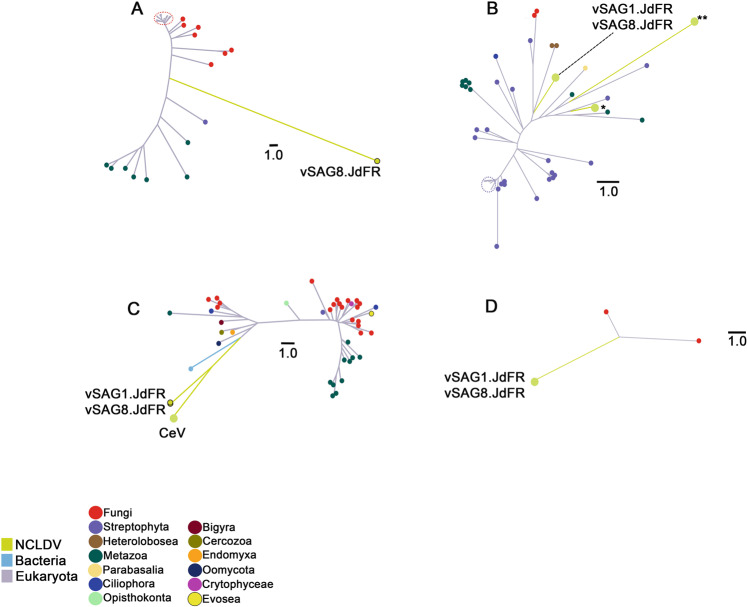
Horizontally transferred genes. Phylogenetic analysis (unrooted trees) of vSAG genes (NCLDVs are green branches). **A** vSAG8.JdFR gene_262, iron-sulfur clusters transporter atm1, mitochondrial enzyme; (**B**) vSAG1.JdFR gene_123 and vSAG8.JdFR gene_198, peroxisome_bioproteinsis_factor_10, variant 2. *Marseillevirus LCMAC201 from deep sea sediments. **Hypothetical protein of Megaviridae environmental sample from marine metagenome; (**C**) vSAG1.JdFR gene_173 and vSAG8.JdFR gene_40, ubiquitin-conjugating enzyme E2-24 kDa-like isoform X2. CeV - *Chrysochromulina ericina* virus, CeV-01b (YP_009173309.1); (**D**) vSAG1.JdFR gene_195 and vSAG8.JdFR gene_62, regulatory protein MIG1.

Among the eukaryotic genes, we identified fungal homolog genes in both vSAGs, which encode two posttranslational modification enzymes (ubiquitin-conjugating enzyme, vSAG1.JdFR Gene 190 and vSAG8.JdFR Gene 57; and iron-sulfur transporter atm1-mitochondrial, vSAG8.JdFR Gene 262), a peroxisome bioproteinsis factor 10, variant 2 (vSAG1.JdFR gene 123 and vSAG8.JdFR gene 198), and a regulatory protein MIG1 (vSAG1.JdFR gene 195 and vSAG8.JdFR gene 62) (Table S9). Phylogenetic analysis of the posttranslational system proteins suggests horizontal gene transfer of genes of fungal origin (Fig. 3A, C).

**Fig. 4:**
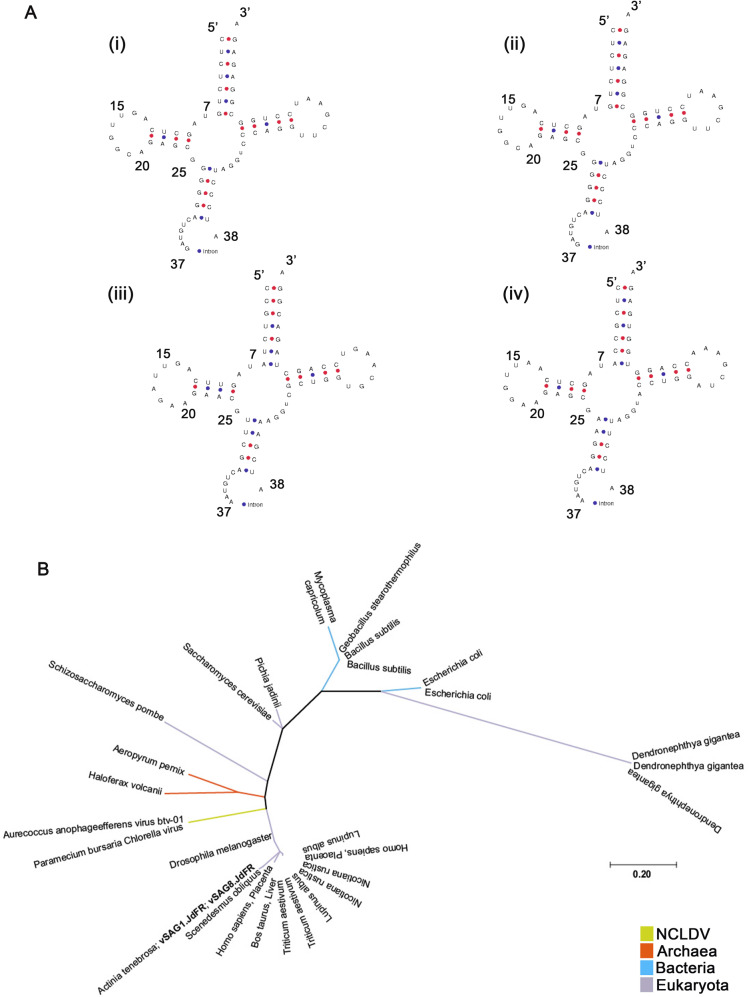
tRNATyr structure and phylogeny. **A** Predicted tyrosine tRNA (tRNATyr) gene structure with intron at the canonical position between nucleotides 37 and 38 of the precursor tRNA **i**. vSAG1.JdFR; **ii**. vSAG8.JdFR; **iii**. *Aureococcus anophagefferens* virus (AaV); **iv**. *Paramecium bursaria Chlorella* virus (PBCV). **B** Phylogenetic position (maximum likelihood tree) of vSAGs based on tRNA^Tyr^ gene.

Additionally, the JdFR vSAGs also contain translation-related genes. The vSAGs have a Tyrosine tRNA (tRNA^Tyr^) gene, which we also found in the *Cafeteria roenbergensis* virus (CroV) (Mimiviridae), *Aureococcus anophagefferens* virus (AaV) (unclassified family-level lineage in the order Imitervirales), Mollivirus sibericum (Pandoraviridae), and *Paramecium bursaria Chlorella* virus (PBCV, *Phycodnaviridae*) (Table S1). Notably, the tRNA^Tyr^ genes in vSAG1.JdFR, vSAG8.JdFR, AaV, and PBCV contain a 20 bps intron at the canonical position between nucleotides 37 and 38 of the precursor tRNA (Fig. 4A). Phylogenic analysis placed the vSAGs’ tRNA^Tyr^ gene (with introns) with eukaryotic tRNA^Tyr^ genes (Fig. 4B). Furthermore, both vSAGs have the eukaryotic translation initiation factor 4E, which is commonly found among Mesomimiviridae and other giant viruses (Fig. S4) [60, 61].

### Fungi, predominantly Ascomycota, are the only confirmed eukaryotes in the crustal fluid samples

Our analysis of the previously published metagenome from Hole U1362B crustal fluids [3, 22] indicated that *Ascomycota* fungi (based on >90% sequence protein homology match) were the dominant eukaryotes in the igneous crust at this borehole (Fig. S5A). *Ascomycota* were also present in the U1362A metagenome [3, 22] in relatively high abundance, although they did not comprise the majority of the identified eukaryotic genes, and protein homology matches were lower than 90% (Fig. S5B). Furthermore, *Ascomycota* taxa were the only eukaryotes that could be confirmed based on nearly complete 18S rRNA gene (Table S2), partially complete 28S rRNA gene (Table S10), and ITS sequences (Table S11) retrieved from the metagenomes. Short 18S rRNA fragments (Table S2) and other eukaryotic gene sequences (Fig. S5A, B) in both metagenomes were affiliated to plants, algae, and animals, none of which are expected to be able to live under the environmental conditions of the igneous crust at this site. While it is possible that eukaryotic environmental DNA that reaches the igneous crust via recharge outcrops is preserved for some time, it is also possible that taxonomic affiliation was skewed by current information in public databases and by the limited information provided by the short sequence fragment’s length.

## Discussion

The two eukaryotic NCLDV draft genomes (vSAG1.JdFR and vSAG8.JdFR) we retrieved from an oceanic crustal fluid sample collected from the IODP borehole U1362B at the Juan de Fuca Ridge were missed in a previously published study of the viral diversity in this environment from samples collected in parallel to our sample from the same borehole and from the nearby borehole U1362A [3]. There were notable methodological differences between how our sample and the samples for the Nigro et al. study [3] were collected and processed. Nigro and colleagues performed metagenomic analyses from large volume (70–124 l) crustal fluid samples collected from Teflon-lined umbilicals and filtered in situ onto 0.22 µm pore-size polyethersulfone filter cartridges. In contrast, we performed single-virus genomics on particles sorted from one milliliter of water collected with a syringe sampler from the freely-venting opening of the borehole observatory. However, geochemical analyses showed consistency between the 64 °C anoxic crustal fluid samples of both studies (Table S12 and references [3, 20, 22]), supporting that the NCLDV particles originated from the crustal fluids, not from possible bottom seawater contamination during sampling. Important, yet possibly numerically rare, NCLDVs could have been missed due to relatively low metagenome sequencing depth, while our targeted method was better suited to detect relatively large virions with large dsDNA genomes.

While the presence of putative bacterial, archaeal, and eukaryotic genes in our vSAGs is not a novel feature for NCLDVs, it provides evidence about their origin from the oceanic basement and points toward fungi as their likely natural hosts. In addition to a core of 10–30 genes acquired mainly by vertical inheritance from ancient ancestors [62], NCLDVs contain multiple genes laterally exchanged between viruses and hosts (current and ancestral), other viruses, or other cellular sources. In either case, the origin of such genes appears to be linked to the host’s ecological niches, with the host providing the grounds for interaction and gene exchange between its viruses, prey, and symbionts [62]. The idea that NCLDVs exist in the crustal fluids with *in situ* hosts that interact with prey or symbiotic bacteria and archaea is further supported by the fact that, as for other NCLDVs, the bacterial and archaeal genes in our vSAGs are clustered in islands suggesting concomitant inheritance [63]. Also, most of their archaeal genes are affiliated to Euryarchaeota present in Juan de Fuca crustal fluids, but not bottom seawater [22].

The presence of translation-related genes, a universal feature for cellular life, in the Juan de Fuca Ridge NCLDVs is also not novel. For example, in this study we found that our vSAGs, CroV, AaV, PBCV, and Mollivirus sibericum have a tRNA^Tyr^ gene. Translational related genes are common in NCDLV, possibly piecemeal acquired from their eukaryotic hosts [18, 64]. Introns at the canonical position between nucleotides 37 and 38 of the precursor tRNA are common for the majority of bacterial, plastid, eukaryotic, and archaeal tRNA introns [65]. However, the short length (20 bps) of our vSAGs’ introns, which is within the range of tRNA introns in eukaryotes and archaea (6–133 bps [65]), and the phylogenic placement of the vSAGs’ tRNA^Tyr^ genes (Fig. 4) suggest a eukaryotic origin.

All the known NCLDVs have eukaryotic hosts, and the evidence we presented above also support that is the case for the Juan de Fuca NCLDVs. We pose that Ascomycota fungi, the dominant (maybe the only) eukaryotes found in the oceanic crust ([66–70], ^this study^), are the likely natural hosts for these viruses, making these viruses the exception to the rule, since all the fungal viruses recognized by the International Committee on Taxonomy of Viruses have RNA genomes, with the exception of ssDNA viruses in the family *Genomoviridae* and two retrotransposons [71]. Those types of fungal virus particles may exist within the oceanic crust; however, our sorting approach was suboptimal to detect them because of inherent limitations of flow cytometry to detect very small particles and the low sensitivity of the fluorescence dye SYBR Green I for RNA and ssDNA. Evidence supporting the hypothesis that the Juan de Fuca NCLDVs infect fungi includes co-occurrence of the viruses with the putative Ascomycota hosts, and the observation that vSAG1.JdFR and vSAG8.JdFR carry fungal genes co-localized in scaffolds with NCLDV genes. In addition, homolog fungal genes (over 70% sequence alignment) in our vSAGs encode two posttranslational modification enzymes (ubiquitin conjugating enzyme, and iron-sulfur transporter atm1-mitochondrial), a peroxisome bioproteinsis factor 10, variant 2, and a regulatory protein MIG1. Phylogenetic placement of those genes suggests a fungal origin, possibly via horizontal gene transfer (HGT), for the two posttranslational modification genes. Prior HGT network analyses also suggested fungi as putative hosts for Nucleocytoviricota klosneuviruses [9]. Additionally, the presence of core NCLDV genes D5-like primase-helicase in the fungus *Allomyces macrogynus* (*Blastocladiomycota* phylum), and B-family DNA polymerase in *Rhizophagus irregularis* (*Mucoromycota* phylum) and *Gonopodaya prolifera* (*Chytridiomycota* phylum) further supports the idea that NCLDVs may infect members of at least some ancestral fungal lineages [57]. Additional work is needed to expand the genomic catalog of NCLDV-host model systems, with a particular focus on fungi.

Fossil records and metagenomic data suggest that active anaerobic fungi may play important ecological and biogeochemical roles in the oceanic crust, where they might have originated as far as 2.4 Ga [66–70, 72, 73]. Recently, a live fungal strain from the genus *Exophiala* (phylum *Ascomycota*) was isolated from the igneous crust at North Pond on the western flank of the Mid-Atlantic Ridge [68, 74]. A study from the lower oceanic crust at Atlantis Bank Gabbro Massif, Indian Ocean revealed over 50% of fungal amplicon sequence variants were from the phylum *Ascomycota* [68]. Our discovery, facilitated by dsDNA-targeted single-virus genomics, of the presumed NCLDVs of fungi adds support to increasing evidence that fungi are active and ecologically important in the oceanic crust habitat [66–70, 72, 73]. Our findings of bacterial and archaeal genes in the vSAGs also support the hypothesis of a symbiotic-like association among fungi and chemoautotrophic archaea and bacteria in subseafloor basalts, where the prokaryotes may use the mycelia for growth while providing a source of carbohydrates or other nutrients to the fungi, as suggested elsewhere [70, 75]. Additionally, H_2_ produced by the fungi in the anoxic environment could serve as an energy source for H_2_-dependent chemoautotrophs such as sulfate-reducing bacteria [66, 69]. Such close physical relationships may allow lateral gene transfer between possible symbionts and the NCLDVs during infection of a putative fungal host. Finally, our study raises important questions regarding the extent of the impact that NCLDV infection of fungi has on productivity and biogeochemical cycling in the marine deep biosphere. Typically, mycoviruses cause persistent infections transmitted intracellularly that do not lyse the host cell [76]. However, not all such infections are asymptomatic. Some mycoviruses induce phenotypic changes that positively or negatively impact host growth, sporulation and virulence [77–80]. However, fungal associations with co-occurring microbes and co-infections [81] by more than one virus type complicate discerning the magnitude and nature of the interactions, i.e., synergistic, antagonistic, or mutualistic.

## Supplementary information

Supplementary Tables and Figures_Legends

Table S1

Table S2

Table S3

Table S4

Table S5

Table S6

Table S7

Table S8

Table S9

Table S10

Table S11

Table S12

Figure S1

Figure S2

Figure S3

Figure S4

Figure S5

## Data Availability

The sequence data for vSAG1.JdFR and vSAG8.JdFR are available through the National Center for Biotechnology Information (NCBI) GenBank Sequence Read Archive under sample accession numbers SRX3120357 and SRX 3120352, respectively, under BioProject PRJNA398661. Annotations of vSAG1.JdFR and vSAG8.JdFR have been submitted to GenBank, NCBI and assigned accession numbers OP765507, and OP765584, respectively. This information is also linked at the Biological and Chemical Oceanography Data Management Office (BCO-DMO) database under dataset ID 717763. Sequence analysis scripts and data can be accessed on github: (https://github.com/asbhattacharjee/NCLDV-from-Juan-de-Fuca-Ridge-flank/blob/main/tRNA_analysis/tRNA_phylogenetic_tree.nwk).
