# Supplementary material for: Genomics discovery of giant fungal viruses from subsurface oceanic crustal fluids
Source: ISME Commun. 2023 Feb 3;3:10. doi: 10.1038/s43705-022-00210-8 (PMC9894930; doi:10.1038/s43705-022-00210-8)
Supplement: Supplementary file 1 — Supplementary Tables and Figures_Legends [file 43705_2022_210_MOESM1_ESM.docx]

**Supplementary Information:**

**Tables**

Table S1: Translational RNA (tRNA) of viruses of Mesomimiviridae subfamily, Paramecium bursaria Chlorella virus (PBCV, Phycodnaviridae), and unclassified giant virus Mollivirus sibericum. The Aurecoccus anophagefferens virus btv-01, Paramecium bursaria Chlorella virus, vSAG1.JdFR, and vSAG8.JdFR have a Tyrosine tRNA (tRNATyr) gene with introns at the position between nucleotides 37 and 38 of the precursor tRNA.

Table S2: Putative hosts of vSAG1.JdFR and vSAG8.JdFR. Eukaryotic 18SrRNA sequences recovered from two metagenome from Juan de Fuca Ridge borehole.

Table S3: Read counts from sorted virus-like particle libraries.

Table S4: De novo assembly statistics and affiliation of two vSAGs.

Table S5: Ancestral NVCOG (clusters) of vSAG1.JdFR and vSAG8.JdFR.

Table S6: List of genes matching archaea: vSAG1.JdFR and vSAG8.JdFR.

Table S7: List of genes matching Bacteria: vSAG1.JdFR and vSAG8.JdFR.

Table S8: List of genes matching Eukaryote: vSAG1.JdFR and vSAG8.JdFR.

Table S9: List of vSAG1.JdFR and vSAG8.JdFR genes acquired from eukaryotes via horizontal gene transfer.

Table S10: 28S rRNA gene sequences recovered from two assembled metagenome from IODP boreholes U1368A and U1362B at the Juan de Fuca Ridge.

Table S11: Internal Transcribe Spacer (ITS) sequences recovered from the assembled metagenome from IODP borehole U1362B at the Juan de Fuca Ridge.

Table S12: Concentration of major and minor elements in fluid samples collected from the Hole U1362B CORK on the Juan de Fuca Ridge flank in comparison to bottom seawater. Chemical comparison of Hole U1362B CORK fluids collected via syringe sampler from fluids freely venting from the top of the open CORK (this study) and via in situ pumping/filtration of fluids through the Tefzel umbilical (sample SSF18 in (3) and (74)) to background bottom seawater (BSW) at this location (shows that the two methods of collecting crustal fluids resulted in nearly identical sample quality. Data in this study provided courtesy of Geoff Wheat, University of Alaska Fairbanks.

**Figure**

Figure S1: **Sorting and multiple displacement amplification (MDA). A**: Cytogram showing virus-like and cellular microbial populations from JdFR borehole U1362B discriminated based on green fluorescence and ice scatter signals. Actual sorting gates are shown as black polygons. The sorted particles and cells are shown individual dots within the scatter plots in the right hand side panels**; B:** MDA kinetics of bacterial/archaeal single-amplified genomes (SAGs) and viral single-amplified genomes (vSAGs). Crossing point (Cp) values represent the number of hours required for each reaction to reach half of the maximum fluorescence level. All MDA products were screened by PCR with archaeal and bacterial specific primers. Grey circles denote positive archaeal primer amplification and black circles denote positive bacterial primer amplification. The amplicons were sequenced to identify the phylogenetic affiliation of the sorted cell. The vSAGs from wells A19, A23, C16, F14, F16, F17, I20, M23, N22, and P22 were sequenced. In this study we identified the draft genomes from the F14 and C16 wells as NCLDVs.

Figure S2: **Phylogenetic affiliations of archaeal and bacterial SAGs from JdFR borehole U1362A and U1362B. A**: Phylogenetic tree of archaea from U1362A and U1362B metagenomes (JdFR-labels) (4) with SAGs from the present study. **B**: Phylogenetic tree of bacteria from U1362A and U1362B metagenomes (JdFR-labels) (4) with SAGs from the present study.

Figure S3: P**hyletic affiliations of the genes from Juan de Fuca Ridge metagenome**. **A:** The bar plot corresponds to the fraction of distinct genes from the borehole U1362B JdFR metagenome classified based on homolog family affiliations to NCLDV. The analysis was based on best blast hits of >30% identity match; **B:** The hierarchal box plot corresponds to the number of genes affiliated to dsDNA viruses from borehole U1362B JdFR metagenome.

Figure S4: **Phylogenetic analysis of vSAGs’ translation system component** Eukaryotic initiation factor 4E. The vSAG1.JdFR and vSAG8.JdFR are in red.

Figure S5: **Relative abundance of genes affiliated to eukaryotes from Juan de Fuca Ridge metagenomes U1362A and U1362B.** **A:** Phyletic affiliations based on best blast hits (>90% similarity) for individual genes from the U1362B borehole metagenome; **B:** Phyletic affiliations of genes in the U1362A borehole metagenome based on best blast hits (>60% but less than <90% identity, no hits had >90% identity).
