# Supplementary material for: Genomics discovery of giant fungal viruses from subsurface oceanic crustal fluids
Source: ISME Commun. 2023 Feb 3;3:10. doi: 10.1038/s43705-022-00210-8 (PMC9894930; doi:10.1038/s43705-022-00210-8)
Supplement: Supplementary file 2 — Table S1 [file 43705_2022_210_MOESM2_ESM.docx]

Table S1: Translational RNA (tRNA) of viruses of Mesomimiviridae subfamily, *Paramecium bursaria Chlorella* virus (PBCV, *Phycodnaviridae*), and unclassified giant virus Mollivirus sibericum. The *Aurecoccus anophagefferens* virus btv-01, *Paramecium bursaria Chlorella* virus, vSAG1.JdFR, and vSAG8.JdFR have a Tyrosine tRNA (tRNA^Tyr^) gene with introns at the position between nucleotides 37 and 38 of the precursor tRNA.

|  | **Sequence Name** | **tRNA #** | **tRNA Begin** | **tRNA End** | **tRNA Type** | **Anticodon** | **Intron Begin** | **Intron End** | **Infernal Score** | **Note** |
| --- | --- | --- | --- | --- | --- | --- | --- | --- | --- | --- |
| **vSAG1.JdFR** | **Genomes from current study** | 1 | 20672 | 20764 | **Tyr** | GTA | **20709** | **20728** | 68.8 | None |
| **vSAG8.JDFR** | **Genomes from current study** | 1 | 20161 | 20253 | **Tyr** | GTA | **20198** | **20217** | 72.3 | None |
| **Aureococcus anophageefferens virus btv-01** | NC_024697 | 1 | 322252 | 322333 | Leu | TAA | 0 | 0 | 52.9 | None |
|  | NC_024697 | 2 | 322372 | 322445 | Ile | TAT | 0 | 0 | 58.1 | None |
|  | NC_024697 | 3 | 322481 | 322585 | Asn | GTT | 0 | 0 | 43.5 | pseudo |
|  | NC_024697 | 4 | 322625 | 322709 | Ser | TGA | 0 | 0 | 50.8 | None |
|  | NC_024697 | 5 | 322727 | 322812 | **Tyr** | GTA | **322764** | **322776** | 54.9 | None |
|  | NC_024697 | 6 | 148390 | 148317 | Arg | TCT | 0 | 0 | 59 | None |
|  | NC_024697 | 7 | 118395 | 118324 | Thr | AGT | 0 | 0 | 57.7 | None |
| **Pandoravirus salinus** | NC_022098 | 1 | 84511 | 84585 | Pro | CGG | 0 | 0 | 71.6 | None |
|  | NC_022098 | 2 | 1812784 | 1812856 | Met | CAT | 0 | 0 | 60.6 | None |
|  | NC_022098 | 3 | 1665818 | 1665746 | Trp | CCA | 0 | 0 | 71.9 | None |
| **Pandoravirus inopinatum** | NC_026440 | 1 | 2166944 | 2166875 | Pro | CGG | 0 | 0 | 38.7 | None |
| **Mollivirus sibericum-P1084** | KR921745 | 1 | 249640 | 249723 | Leu | CAA | 0 | 0 | 75.5 | None |
|  | KR921745 | 2 | 618519 | 618447 | Met | CAT | 0 | 0 | 63.4 | None |
|  | KR921745 | 3 | 65090 | 65016 | **Tyr** | GTA | 0 | 0 | 35.7 | None |
| **Tetraselmis virus 1** | KY322437 | 1 | 372744 | 372817 | Ile | TAT | 0 | 0 | 76.8 | None |
|  | KY322437 | 2 | 372827 | 372899 | Lys | TTT | 0 | 0 | 81 | None |
|  | KY322437 | 3 | 372926 | 373009 | Leu | TAA | 0 | 0 | 68.2 | None |
|  | KY322437 | 4 | 373015 | 373087 | Lys | TTT | 0 | 0 | 79.8 | None |
|  | KY322437 | 5 | 373124 | 373204 | Leu | CAA | 0 | 0 | 66 | None |
|  | KY322437 | 6 | 373207 | 373279 | Glu | TTC | 0 | 0 | 63.3 | None |
|  | KY322437 | 7 | 373290 | 373362 | Arg | TCT | 0 | 0 | 73.2 | None |
|  | KY322437 | 8 | 373385 | 373458 | Asn | GTT | 0 | 0 | 80.5 | None |
|  | KY322437 | 9 | 373460 | 373533 | Ile | TAT | 0 | 0 | 76.8 | None |
|  | KY322437 | 10 | 373548 | 373621 | Asn | GTT | 0 | 0 | 81.3 | None |
| **Phaeocycstis globosa virus 12T** | HQ634147 | 1 | 177016 | 177095 | Leu | CAA | 0 | 0 | 37.8 | None |
|  | HQ634147 | 2 | 409258 | 409331 | Ile | TAT | 0 | 0 | 69.3 | None |
|  | HQ634147 | 3 | 409342 | 409413 | Gln | TTG | 0 | 0 | 56.3 | None |
|  | HQ634147 | 4 | 409419 | 409492 | Asn | GTT | 0 | 0 | 74.2 | None |
|  | HQ634147 | 5 | 409520 | 409603 | Leu | TAA | 0 | 0 | 57.1 | None |
|  | HQ634147 | 6 | 409609 | 409682 | Asn | GTT | 0 | 0 | 74.2 | None |
|  | HQ634147 | 7 | 409705 | 409788 | Leu | TAA | 0 | 0 | 62.6 | None |
|  | HQ634147 | 8 | 410799 | 410871 | Arg | TCT | 0 | 0 | 67.7 | None |
| **Chrysochromulina ericina virus cev-01b** | NC_028094 | 1 | 235797 | 235879 | Ser | TGA | 0 | 0 | 68.8 | None |
|  | NC_028094 | 2 | 235890 | 235962 | Ala | TGC | 0 | 0 | 51.6 | None |
|  | NC_028094 | 3 | 235969 | 236051 | Ser | GCT | 0 | 0 | 70.5 | None |
|  | NC_028094 | 4 | 423376 | 423449 | Ile | TAT | 0 | 0 | 66.9 | None |
|  | NC_028094 | 5 | 423494 | 423566 | Lys | TTT | 0 | 0 | 35.2 | None |
|  | NC_028094 | 6 | 423572 | 423643 | Gln | TTG | 0 | 0 | 59.4 | None |
|  | NC_028094 | 7 | 423766 | 423847 | Leu | TAA | 0 | 0 | 69.8 | None |
|  | NC_028094 | 8 | 423964 | 424037 | Asn | GTT | 0 | 0 | 73.9 | None |
|  | NC_028094 | 9 | 424072 | 424144 | Lys | TTT | 0 | 0 | 72 | None |
|  | NC_028094 | 10 | 424179 | 424253 | Arg | TCT | 0 | 0 | 70.1 | None |
|  | NC_028094 | 11 | 383764 | 383694 | Gly | TCC | 0 | 0 | 64.2 | None |
|  | NC_028094 | 12 | 74561 | 74480 | Leu | CAA | 0 | 0 | 68.1 | None |
| **Phaeocycstis globosa virus 16T** | KC662249 | 1 | 176759 | 176838 | Leu | CAA | 0 | 0 | 37.8 | None |
|  | KC662249 | 2 | 408944 | 409017 | Ile | TAT | 0 | 0 | 69.3 | None |
|  | KC662249 | 3 | 409028 | 409099 | Gln | TTG | 0 | 0 | 56.3 | None |
|  | KC662249 | 4 | 409105 | 409178 | Asn | GTT | 0 | 0 | 74.2 | None |
|  | KC662249 | 5 | 409207 | 409290 | Leu | TAA | 0 | 0 | 57.1 | None |
|  | KC662249 | 6 | 409296 | 409369 | Asn | GTT | 0 | 0 | 74.2 | None |
|  | KC662249 | 7 | 409393 | 409476 | Leu | TAA | 0 | 0 | 62.6 | None |
|  | KC662249 | 8 | 410487 | 410559 | Arg | TCT | 0 | 0 | 67.7 | None |
| **Paramecium bursaria chlorella virus** | DQ491001 | 1 | 134513 | 134585 | Ile | TAT | 0 | 0 | 63.3 | None |
|  | DQ491001 | 2 | 134610 | 134693 | Leu | TAA | 0 | 0 | 51.7 | None |
|  | DQ491001 | 3 | 134718 | 134790 | Phe | GAA | 0 | 0 | 57.1 | None |
|  | DQ491001 | 4 | 134814 | 134889 | Arg | TCT | 0 | 0 | 73.1 | None |
|  | DQ491001 | 5 | 134890 | 134960 | Gly | TCC | 0 | 0 | 57.3 | None |
|  | DQ491001 | 6 | 134984 | 135058 | Asn | GTT | 0 | 0 | 74.6 | None |
|  | DQ491001 | 7 | 135080 | 135152 | Asn | GTT | 0 | 0 | 49.9 | None |
|  | DQ491001 | 8 | 135174 | 135259 | **Tyr** | GTA | **135211** | **135223** | 47.1 | None |
|  | DQ491001 | 9 | 135262 | 135334 | Lys | CTT | 0 | 0 | 86.5 | None |
|  | DQ491001 | 10 | 135496 | 135567 | Thr | CGT | 0 | 0 | 77.8 | None |
| **Cafeteria roenbergensis virus bv-pw1** | NC_014637 | 1 | 509015 | 509086 | **Tyr** | GTA | 0 | 0 | 68.1 | None |
|  | NC_014637 | 2 | 509181 | 509265 | Leu | TAA | 0 | 0 | 61.3 | None |
|  | NC_014637 | 3 | 509266 | 509333 | Undet | NNN | 0 | 0 | 30.2 | None |
|  | NC_014637 | 4 | 509421 | 509502 | Leu | TAA | 0 | 0 | 58.7 | None |
|  | NC_014637 | 5 | 509506 | 509583 | Lys | TTT | 0 | 0 | 72.5 | None |
|  | NC_014637 | 6 | 509587 | 509658 | Sup | TTA | 0 | 0 | 48.9 | None |
|  | NC_014637 | 7 | 509911 | 509992 | Leu | TAA | 0 | 0 | 61.3 | None |
|  | NC_014637 | 8 | 510066 | 510135 | **Tyr** | ATA | 0 | 0 | 34.9 | None |
|  | NC_014637 | 9 | 510177 | 510261 | Leu | TAA | 0 | 0 | 61.3 | None |
|  | NC_014637 | 10 | 510262 | 510329 | Undet | NNN | 0 | 0 | 26.3 | None |
|  | NC_014637 | 11 | 510417 | 510501 | Leu | TAA | 0 | 0 | 58.7 | None |
|  | NC_014637 | 12 | 510502 | 510569 | Undet | NNN | 0 | 0 | 28.4 | None |
|  | NC_014637 | 13 | 510656 | 510737 | Leu | TAA | 0 | 0 | 58.7 | None |
|  | NC_014637 | 14 | 510741 | 510818 | Lys | TTT | 0 | 0 | 73.5 | None |
|  | NC_014637 | 15 | 511091 | 511175 | Leu | TAA | 0 | 0 | 61.3 | None |
|  | NC_014637 | 16 | 511176 | 511243 | Undet | NNN | 0 | 0 | 26.3 | None |
|  | NC_014637 | 17 | 511330 | 511414 | Leu | TAA | 0 | 0 | 58.7 | None |
|  | NC_014637 | 18 | 511415 | 511482 | Undet | NNN | 0 | 0 | 28.4 | None |
|  | NC_014637 | 19 | 511570 | 511651 | Leu | TAA | 0 | 0 | 58.7 | None |
|  | NC_014637 | 20 | 511655 | 511732 | Lys | TTT | 0 | 0 | 73.5 | None |
|  | NC_014637 | 21 | 511736 | 511809 | Asn | GTT | 0 | 0 | 59.9 | None |
| **Organic Lake phycodnavirus 1** | OLPV 1 | 0 | 0 | 0 | 0 | 0 | 0 | 0 | 0 | None |
| **Organic Lake**  **phycodnavirus 2** | OLPV 2 | 0 | 0 | 0 | 0 | 0 | 0 | 0 | 0 | None |
