# Supplementary material for: Genomics discovery of giant fungal viruses from subsurface oceanic crustal fluids
Source: ISME Commun. 2023 Feb 3;3:10. doi: 10.1038/s43705-022-00210-8 (PMC9894930; doi:10.1038/s43705-022-00210-8)
Supplement: Supplementary file 3 — Table S2 [file 43705_2022_210_MOESM3_ESM.docx]

Table S2: Putative hosts of vSAG1.JdFR and vSAG8.JdFR. Eukaryotic 18SrRNA sequences recovered from two metagenome from Juan de Fuca Ridge borehole.

| **GeneID** | **Length (bps)** | **Identity (Silva)** | **Taxonomy** | **Copy number (JGI)** |
| --- | --- | --- | --- | --- |
| JGI24019J35510_10078192 | 1750 | 99.74% | *Eukaryota; Fungi; Dikarya;* ***Ascomycota****; Pezizomycotina;*  *Eurotiomycetes; Eurotiomycetidae; Eurotiales; Aspergillaceae;* | 86 |
| JGI24019J35510_10124471 | 1244 | 99.84% | *Eukaryota; Fungi; Dikarya;* ***Ascomycota****; Pezizomycotina;*  *Sordariomycetes; Hypocreomycetidae; Hypocreales; Cordycipitaceae;* | 47 |
| **Partial 18S rRNA** | | | | |
| JGI24019J35510_1006172 | 502 | 100% | *Eukaryota; Fungi;Dikarya;****Ascomycota****;Pezizomycotina;* | 28 |
| JGI24019J35510_10213491 | 611 | 99.50% | *Eukaryota; Fungi;Dikarya;****Ascomycota****;Pezizomycotina;Eurotiomycetes;*  *Eurotiales;Aspergillaceae;Aspergillus;* | 44 |
| JGI24019J35510_10804581 | 402 | 100% | *Eukaryota; Metazoa (Animalia);Eumetazoa;Bilateria;Chordata;*  *Vertebrata;Gnathostomata;Euteleostomi;Tetrapoda;Mammalia;* | 23 |
| JGI24019J35510_10981471 | 357 | 69.35% | *Unclassified;* | 9 |
| JGI24019J35510_11179141 | 324 | 99.69% | *Eukaryota; Metazoa (Animalia);Eumetazoa;Bilateria;Chordata;Vertebrata;*  *Gnathostomata;Euteleostomi;Tetrapoda;Mammalia;* | 7 |
| JGI24019J35510_11204312 | 75 | 100% | *Eukaryota;Fungi;Dikarya;****Ascomycota****;Pezizomycotina;Eurotiomycetes;*  *Eurotiales;Aspergillaceae;Aspergillus;* | 18 |
| JGI24019J35510_11261321 | 270 | 98.14% | *Eukaryota;Archaeplastida;Chloroplastida;Charophyta;Phragmoplastophyta;*  *Streptophyta;Embryophyta;Tracheophyta;Spermatophyta;Magnoliophyta;Brassicales;* | 13 |
| JGI24019J35510_11465141 | 88 | 66.21% | *Unclassified;* | 9 |
| JGI24019J35510_11619981 | 204 | 100% | *Eukaryota; Metazoa (Animalia);Eumetazoa;Bilateria;Chordata;Vertebrata;*  *Gnathostomata;Euteleostomi;Tetrapoda;Mammalia;* | 8 |
| JGI24019J35510_11890531 | 256 | 100% | *Eukaryota;Excavata;Discoba;Discicristata;Heterolobosea;Tetramitia;Vahlkampfia;* | 12 |
| JGI24019J35510_11998001 | 246 | 49.45 | *Unclassified;* | 6 |

^*^Zero 18SrRNA gene sequences were recovered from assembled metagenome from IODP boreholes U1362B at the Juan de Fuca Ridge
