# Supplementary material for: Genomics discovery of giant fungal viruses from subsurface oceanic crustal fluids
Source: ISME Commun. 2023 Feb 3;3:10. doi: 10.1038/s43705-022-00210-8 (PMC9894930; doi:10.1038/s43705-022-00210-8)
Supplement: Supplementary file 4 — Table S3 [file 43705_2022_210_MOESM4_ESM.docx]

Table S3: Read counts from sorted virus-like particle libraries.

| **Library name** | **JGI Project** | **vSAG name** | **# of raw reads**  **(PE)** | **# of quality filtered reads (PE)** | **# post removal of low complexity reads (PE)^$^** | **# of reads used in SPAdes assembly (PE) ^$^** |
| --- | --- | --- | --- | --- | --- | --- |
| AAUAN, TNNY^*^^ | 1031158 | vSAG1.JdFR | 18,408,697 | 18,061,647 | 17,976,119 | 17,932,701 |
| AAUAH^¶^ | 1031154 | vSAG8.JdFR | 14,123,402 | 14,052,213 | 5,410,851 | 5,361,576 |

^*^ Additional library prepared and sequenced from a separate MDA aliquot.

^$^ Interleaved reads.

^ NCBI Bioproject # PRJNA398661; SRA # SRX3120357; Biosample # SAMN075115454; GenBank Accession number OP765507.

^¶^ NCBI Bioproject # PRJNA398661; SRA # SRX3120352; Biosample # SAMN075115453; GenBank accession number OP765584.
