# Supplementary material for: Genomics discovery of giant fungal viruses from subsurface oceanic crustal fluids
Source: ISME Commun. 2023 Feb 3;3:10. doi: 10.1038/s43705-022-00210-8 (PMC9894930; doi:10.1038/s43705-022-00210-8)
Supplement: Supplementary file 5 — Table S4 [file 43705_2022_210_MOESM5_ESM.docx]

Table S4: *De novo* assembly statistics and affiliation of two vSAGs.

| **Library name** | **JGI Project** | **vSAG name** | **# of scaffolds / Size (bps)** | **Largest scaffold (bp)** | **N50 (bp)** | **%GC** | **# of CDS**^!^ | **Affiliation phylum^$^** | **# of tRNAs^&^** |
| --- | --- | --- | --- | --- | --- | --- | --- | --- | --- |
| AAUAN, TNNY^*^^ | 1031158 | vSAG1.JdFR | 28 / 267,752 | 65,366 | 14,923 | 21.9 | 277 | Nucleocytoviricota | 1 |
| AAUAH^¶^ | 1031154 | vSAG8.JdFR | 33 / 300,125 | 60,221 | 33,363 | 21.2 | 302 | Nucleocytoviricota | 1 |

^*^ Additional library prepared and sequenced from a separate MDA aliquot.

N50: The minimum contig length needed to cover 50% of the genome

^!^ Coding sequences

^$^ Based on blastp analysis (evalue 1e-05) against NCBI viral reference sequence database as of January 2018.

^&^ tRNAScan-SE prediction identified as tRNA^Tyr^.

^ NCBI Bioproject # PRJNA398661; SRA # SRX3120357; Biosample # SAMN075115454; GenBank accession number OP765507.

^¶^ NCBI Bioproject # PRJNA398661; SRA # SRX3120352; Biosample # SAMN075115453; GenBank accession number OP765584.
