# Supplementary material for: Genomics discovery of giant fungal viruses from subsurface oceanic crustal fluids
Source: ISME Commun. 2023 Feb 3;3:10. doi: 10.1038/s43705-022-00210-8 (PMC9894930; doi:10.1038/s43705-022-00210-8)
Supplement: Supplementary file 6 — Table S5 [file 43705_2022_210_MOESM6_ESM.docx]

Table S5: Ancestral NVCOG (clusters) of vSAG1.JdFR and vSAG8.JdFR

| **Cluster name** | **Functional category** | **Cluster annotation** | **Cluster presence & absence** | |
| --- | --- | --- | --- | --- |
|  |  |  | **vSAG1.JdFR** | **vSAG8.JdFR** |
| NCVOG0004 | DNA replication, recombination and repair | AP (apurinic) endonuclease family 2 - bacterial | N | N |
| NCVOG0022 | Virion structure and morphogenesis | NCLDV major capsid protein (pfam03340 for Poxviridae; pfam04451 for others) | Y | Y |
| NCVOG0023 | DNA replication, recombination and repair | D5-like helicase-primase | N | N |
| NCVOG0035 | DNA replication, recombination and repair | NAD+ dependent DNA ligase (smart00532) | N | N |
| NCVOG0036 | DNA replication, recombination and repair | DNA topoisomerase I | N | N |
| NCVOG0038 | DNA replication, recombination and repair | DNA polymerase elongation subunit family B | Y | Y |
| NCVOG0052 | Other metabolic functions | disulfide (thiol) oxidoreductase; Erv1 / Alr family (pfam04777) | Y | Y |
| NCVOG0059 | Other metabolic functions | FtsJ-like methyltransferase family proteins (pfam01728) | Y | Y |
| NCVOG0076 | DNA replication, recombination and repair | DNA or RNA helicases of superfamily II (COG1061) | Y | Y |
| NCVOG0211 | Virion structure and morphogenesis | myristylated IMV envelope protein (pfam02442: Lipid membrane protein of large eukaryotic DNA viruses) | N | N |
| NCVOG0249 | Virion structure and morphogenesis | A32-like packaging ATPase | Y | Y |
| NCVOG0256 | Other metabolic functions | IMV envelope protein p35 | Y | Y |
| NCVOG0262 | DNA replication, recombination and repair | RNA-helicase DExH-NPH-II | N | Y |
| NCVOG1060 | DNA replication, recombination and repair | FLAP-like endonuclease XPG (cd00128) | N | N |
| NCVOG1088 | Transcription and RNA processing | RNA ligase (conserved in irido-, asfa- asco- and Marseille viruses) | Y | Y |
| NCVOG1115 | Other metabolic functions | uracil-DNA glycosylase | N | Y |
| NCVOG1117 | Transcription and RNA processing | mRNA capping enzyme large subunit | Y | Y |
| NCVOG1122 | Virion structure and morphogenesis | Myristylated protein; pfam03003, DUF230 | N | N |
| NCVOG1127 | Transcription and RNA processing | transcription initiation factor IIB | Y | Y |
| NCVOG1192 | DNA replication, recombination and repair | YqaJ viral recombinase family: pfam09588: This protein family is found in many different bacterial species but is of viral origin | Y | Y |
