# Supplementary material for: Genomics discovery of giant fungal viruses from subsurface oceanic crustal fluids
Source: ISME Commun. 2023 Feb 3;3:10. doi: 10.1038/s43705-022-00210-8 (PMC9894930; doi:10.1038/s43705-022-00210-8)
Supplement: Supplementary file 7 — Table S6 [file 43705_2022_210_MOESM7_ESM.docx]

Table S6: List of genes matching archaea: vSAG1.JdFR and vSAG8.JdFR

| **GeneID** | **%ID** | **E-Value [vSAG1.JdFR]** | **E-Value [vSAG8.JdFR]** | **Bitscore [vSAG1.JdFR]** | **Bitscore [vSAG8.JdFR]** | **Annotation** | **Phyletic affiliations** | **Environment** | **Kingdom** | **Phylum** |
| --- | --- | --- | --- | --- | --- | --- | --- | --- | --- | --- |
| Gene 13* Gene 12** | 59.155 | 5.04E-140 | 4.98E-140 | 411 | 411 | GDP-mannose 4,6-dehydratase | *Methanosarcina barkeri* | Sewage | Archaea | Euryarchaeota! |
| Gene 45* Gene 94** | 81.013 | 1.9E-37 | 1.88E-37 | 130 | 130 | Hypothetical protein CXT73 07345 | *Euryarchaeota archaeon* | Hydrothermal plume | Archaea | Euryarchaeota! |
| Gene 53* Gene 110** | 51.572 | 5.42E-50 | 5.35E-50 | 168 | 168 | Hypothetical protein CXT73 03325 | *Euryarchaeota archaeon* | Hydrothermal plume | Archaea | Euryarchaeota! |
| Gene 91* Gene 289** | 40 | 0.00000015 | NA | 62.8 | NA | Hypothetical protein CXT73 07395 | *Euryarchaeota archaeon* | Hydrothermal plume | Archaea | Euryarchaeota! |
| Gene 103* Gene 173** | 55.677 | 0 | 0 | 548 | 548 | Hypothetical protein AKJ59 00295 | *Ca’* division MSBL1 archaeon SCGC-AAA385M02 | Anoxic environment (Brine pool) | Archaea | Euryarchaeota! |
| Gene 104* Gene 174** | 27.219 | 0.000106 | 0.000105 | 50.8 | 50.8 | Hypothetical protein AKJ59 00800 | *Ca’* division MSBL1 archaeon SCGC-AAA385M02 | Anoxic environment (Brine pool) | Archaea | Euryarchaeota! |
| Gene 105* Gene 175** | 32.484 | 8E-37 | 7.94E-37 | 143 | 143 | Hypothetical protein AKJ59 00040 | *Ca’* division MSBL1 archaeon SCGC-AAA385M02 | Anoxic environment (Brine pool) | Archaea | Euryarchaeota! |
| Gene 113* Gene 188** | 45.342 | 2.51E-52 | 2.49E-52 | 176 | 176 | Hypothetical protein CXT73 04500 | *Euryarchaeota archaeon* | Hydrothermal plume | Archaea | Euryarchaeota! |
| Gene 139* | 27.739 | 3.56E-71 | NA | 273 | NA | Hypothetical protein CXT73 06340, partial | *Euryarchaeota archaeon* | Hydrothermal plume | Archaea | Euryarchaeota! |
| Gene 142* | 65.838 | 0 | NA | 2131 | NA | Hypothetical protein CXT73 05625 | *Euryarchaeota archaeon* | Hydrothermal plume | Archaea | Euryarchaeota! |
| Gene 147* Gene 234** | 36.158 | 2.53E-31 | 2.51E-31 | 125 | 125 | Hypothetical protein CXT73 04075 | *Euryarchaeota archaeon* | Hydrothermal plume | Archaea | Euryarchaeota! |
| Gene 153* Gene 228** | 27.855 | 2.22E-29 | 2.21E-29 | 126 | 126 | Hypothetical protein CXT73 03485 | *Euryarchaeota archaeon* | Hydrothermal plume | Archaea | Euryarchaeota! |
| Gene 175* Gene 42** | 32.487 | 3.26E-16 | 3.24E-16 | 83.2 | 83.2 | Hypothetical protein CXT73 06445 | *Euryarchaeota archaeon* | Hydrothermal plume | Archaea | Euryarchaeota! |
| Gene 207* Gene 308** | 50.769 | 1.96E-11 | 1.8E-11 | 65.5 | 65.1 | Hypothetical protein CXT73 05460 | *Euryarchaeota archaeon* | Hydrothermal plume | Archaea | Euryarchaeota! |
| Gene 216* | 43.59 | 1.33E-31 | NA | 145 | NA | DUF5011 domain-containing protein, partial | *Ca’* Nitrosotenuis sp. | Freshwater | Archaea | Thaumarchaeota |
| Gene 226* | 48.276 | 3.28E-10 | NA | 63.5 |  | Hypothetical protein CXT73 06855 | *Euryarchaeota archaeon* | Hydrothermal plume | Archaea | Euryarchaeota! |
| Gene 231* Gene 284** | 32.692 | 0.000816 | 0.000807 | 47.4 | 47.4 | Hypothetical protein AKJ59 00790 | *Ca’* division MSBL1 archaeon SCGC-AAA385M02 | Anoxic environment (Brine pool) | Archaea | Euryarchaeota! |
| Gene 237* Gene 284** | 47.368 | 1.1 | 0.000807 | 36.6 | 47.4 | Hypothetical protein AKJ59 00790 | *Ca’* division MSBL1 archaeon SCGC-AAA385M02 | Anoxic environment (Brine pool) | Archaea | Euryarchaeota! |
| Gene 262* | 48.387 | 1.46E-43 | NA | 155 | NA | Hypothetical protein CXT73 07725 | *Euryarchaeota archaeon* | Hydrothermal plume | Archaea | Euryarchaeota! |
| Gene 269* Gene 285** | 35.496 | 6.9E-33 | 0.000807 | 133 | 47.4 | Hypothetical protein AKJ59 00790 | *Ca’* division MSBL1 archaeon SCGC-AAA385M02 | Anoxic environment (Brine pool) | Archaea | Euryarchaeota! |
| Gene 129** | 56.25 | NA | 2.83E-18 | NA | 92 | Hypothetical protein CXT73 02500 | *Euryarchaeota archaeon* | Hydrothermal plume | Archaea | Euryarchaeota! |
| Gene 155** | 32.432 | NA | 5.45E-10 | NA | 63.2 | Hypothetical protein CXT73 04930 | *Euryarchaeota archaeon* | Hydrothermal plume | Archaea | Euryarchaeota! |
| Gene 169** | 31.008 | NA | 5.55E-19 | NA | 97.8 | Hypothetical protein CXT73 07660 | *Euryarchaeota archaeon* | Hyrothrermal plume | Archaea | Euryarchaeota! |
| Gene 173** | 55.677 | NA | 0 | NA | 548 | Hypothetical protein AKJ59 00295 | *Ca’* division MSBL1 archaeon SCGC-AAA385M02 | Anoxic environment (Brine pool) | Archaea | Euryarchaeota! |
| Gene 238** | 51.493 | NA | 1.7E-136 | NA | 419 | Hypothetical protein CXT73 04100 | *Euryarchaeota archaeon* | Hydrothermal Plume | Archaea | Euryarchaeota! |
| Gene 272** | 32.675 | NA | 3.72E-78 | NA | 265 | Hypothetical protein CXT73 04825, partial | *Euryarchaeota archaeon* | Hydrothermal Plume | Archaea | Euryarchaeota! |
| Gene 276** | 47.368 | NA | 1 | NA | 36.6 | Hypothetical protein AKJ59 00790 | *Ca’* division MSBL1 archaeon SCGC-AAA385M02 | Anoxic environment (Brine pool) | Archaea | Euryarchaeota! |
| Gene 289** | 40 | NA | 0.000000148 | NA | 62.8 | Hypothetical protein CXT73 07395 | *Euryarchaeota archaeon* | Hydrothermal Plume | Archaea | Euryarchaeota! |
| Gene 298** | 34.975 | NA | 2.13E-13 | NA | 75.5 | Hypothetical protein AKJ59 00090 | *Ca’* division MSBL1 archaeon SCGC-AAA385M02 | Anoxic environment (Brine pool) | Archaea | Euryarchaeota! |
| Gene 300** | 35.496 | NA | 6.82E-33 | NA | 133 | Hypothetical protein AKJ59 00790 | *Ca’* division MSBL1 archaeon SCGC-AAA385M02 | Anoxic environment (Brine pool) | Archaea | Euryarchaeota! |

* vSAG1.JdFR

** vSAG8.JdFR

! Genes affiliated to Euryarchaeota

Detailed protein BLAST results are enlisted in vSAG1.JdFR annotation and vSAG8.JdFR annotation excel sheets
