# Supplementary material for: Genomics discovery of giant fungal viruses from subsurface oceanic crustal fluids
Source: ISME Commun. 2023 Feb 3;3:10. doi: 10.1038/s43705-022-00210-8 (PMC9894930; doi:10.1038/s43705-022-00210-8)
Supplement: Supplementary file 8 — Table S7 [file 43705_2022_210_MOESM8_ESM.docx]

Table S7: List of genes matching Bacteria: vSAG1.JdFR and vSAG8.JdFR

| **GeneID** | **%ID** | **E-Value [vSAG1.JdFR]** | **E-Value [vSAG8.JdFR]** | **Bitscore [vSAG1.JdFR]** | **Bitscore [vSAG8.JdFR]** | **Annotation** | **Phyletic Affiliations** | **Environment** | **Kingdom** | **Phylum** |
| --- | --- | --- | --- | --- | --- | --- | --- | --- | --- | --- |
| Gene 4* Gene 3** | 35.079 | 2.03E-17 | 2.01E-17 | 85.9 | 85.9 | Hypothetical protein | *Moorea producens* | Marine | Bacteria | Cyanobacteria |
| Gene 5* Gene 4** | 29.969 | 5.14E-35 | 5.08E-35 | 140 | 140 | Hypothetical protein CKO51 01665 | *Rhodopirellula sp.* SM50 | Beach sediment surface | Bacteria | Planctomycetes |
| Gene 8* Gene7** | 29.268 | 0.088 | 0.087 | 43.1 | 43.1 | Glycoside hydrolase family 30 protein | *Arenitalea lutea* | Intertidal sand | Bacteria | Bacteroidetes |
| Gene 10* Gene 9** | 47.234 | 5.80E-58 | 5.73E-58 | 193 | 193 | Glycosyltransferase | *Shewanella sp.* XMDDZSB0408 | Intestine of Haliotis diversicolor | Bacteria | Proteobacteria |
| Gene 11* Gene 10** | 28.295 | 1.57E-07 | 1.55E-07 | 64.7 | 64.7 | Hypothetical protein GM48 0100 | *Actinobacterium acIB-AMD-7* | Water column of the freshwater reservoir Embalse-de-Amadorio in Spain | Bacteria | Actinobacteria |
| Gene 19* Gene 18** | 36.667 | 2.6 | 2.6 | 37.4 | 37.4 | NADH-quinone oxidoreductase subunit I | *Chryseobacterium haifense* | Psychrotolerant isolated from raw milk | Bacteria | Bacteroidetes |
| Gene 25* Gene 24** | 32.836 | 0.15 | 0.15 | 42.4 | 42.4 | Multidrug RND transporter | *Ca’* division KSB1 bacterium | Zodletone Spring, Anadarko, OK: Low-Salt, Sulfide- and Sulfur-Rich Spring | Bacteria | Unclassified |
| Gene 27* Gene 26** | 52.41 | 2.04E-39 | 2.02E-39 | 153 | 153 | Hypothetical protein CBC22 04935 | *Alphaproteobacteria bacterium* TMED62 | Mediterranean Sea | Bacteria | Proteobacteria |
| Gene 30* Gene 29** | 46.617 | 1.46E-32 | 1.45E-32 | 123 | 123 | Hypothetical protein CBB97 08775 | *Ca’* Endolissoclinum sp. TMED37 | Mediterranean Sea | Bacteria | Proteobacteria |
| Gene 31* Gene 30** | 64.516 | 9.68E-35 | 9.57E-35 | 132 | 132 | Hypothetical protein CBC22 04770 | *Alphaproteobacteria bacterium* TMED62 | Mediterranean Sea | Bacteria | Proteobacteria |
| Gene 32* Gene 31** | 46.602 | 0.003 | 0.003 | 50.4 | 50.4 | DNA translocase FtsK | *Rummeliibacillus sp.* TYF005 | Spoiled Vinegar: high ethanol and salt tolerance | Bacteria | Firmicutes |
| Gene 35* Gene 34** | 42.857 | 3.47E-37 | 3.43E-37 | 137 | 137 | Hypothetical protein CBB97 08810 | *Ca’* Endolissoclinum sp. TMED37 | Mediterranean Sea | Bacteria | Proteobacteria |
| Gene 36* Gene 35** | 40.58 | 0.091 | 0.09 | 41.6 | 41.6 | DNA translocase FtsK, partial | *Bacillus sp*. LB | Soil | Bacteria | Firmicutes |
| Gene 49* Gene 114** | 29.301 | 2.51E-21 | 2.48E-21 | 112 | 112 | Surface protein | *Fibrobacter sp*. UWB13 | Unknown | Bacteria | Fibrobacteres |
| Gene 64* Gene 99** | 58.088 | 1.07E-53 | 1.06E-53 | 175 | 175 | dCMP deaminase | *Rhodobacteraceae bacterium* | Indian Ocean: Eastern Africa Coastal Province: marine water sample | Bacteria | Proteobacteria |
| Gene 87* Gene 153** | 53.846 | 7.1 | 7 | 32.7 | 32.7 | Hypothetical protein PMI41 02489 | *Phyllobacterium sp.* YR531 | Rhizosphere and Endosphere of Populus deltoides | Bacteria | Proteobacteria |
| Gene 89* | 29.293 | 0.023 | NA | 44.3 | NA | PAS domain S-box protein | *Thiocapsa marina* | Brackish to marine sediments in the Mediterranean Sea | Bacteria | Proteobacteria |
| Gene 118* Gene 193** | 38.393 | 9.88E-26 | 9.80E-26 | 111 | 111 | Hypothetical protein | *Geminocystis herdmanii* | Lake water | Bacteria | Cyanobacteria |
| Gene 126* Gene 201** | 29.524 | 0.15 | 0.15 | 43.5 | 43.5 | Glycosyl transferase family 1, partial | *Staphylococcus haemolyticus* | Skin flora of Humans and animals | Bacteria | Firmicutes |
| Gene 129* Gene 204** | 51.724 | 7.2 | 7.1 | 34.7 | 34.7 | Inorganic phosphate transporter | *Ca’* Dactylopiibacterium carminicum | Endosymboiant of Dactylopius coccus | Bacteria | Proteobacteria |
| Gene 158* | 55.153 | 1.80E-137 | NA | 405 | NA | Guanosine monophosphate reductase | *Ca’* Aerophobetes bacterium | Atlantic Ocean: North Pond, marine subsurface aquifer | Bacteria | Candidatus Aerophobetes |
| Gene 186* Gene 53** | 56.643 | 0 | 0 | 660 | 660 | Hypothetical protein CBC22 04870 | *Alphaproteobacteria bacterium* TMED62 | Mediterranean Sea | Bacteria | Proteobacteria |
| Gene 189* Gene 56** | 49 | 9.78E-23 | 9.71E-23 | 97.1 | 97.1 | Hypothetical protein CBC22 04875 | *Alphaproteobacteria bacterium* TMED62 | Mediterranean Sea | Bacteria | Proteobacteria |
| Gene 197* | 31.933 | 1.05E-04 | NA | 57 | NA | Vomp family autotransporter, partial | *Bartonella birtlesii* | Human pathogen | Bacteria | Proteobacteria |
| Gene 199* Gene 268** | 33.198 | 1.19E-18 | 1.18E-18 | 92.4 | 92.4 | Hypothetical protein | *Synechococcus sp*. WH 7803 | Marine | Bacteria | Cyanobacteria |
| Gene 217* | 35.385 | 9.69E-20 | NA | 101 | NA | Leucine-rich repeat domain-containing protein | *Brachyspira hampsonii* | pathogens in pigs, birds, dogs, and humans | Bacteria | Spirochaetes |
| Gene 223* Gene 165** | 21.296 | 1.7 | 1.60E+00 | 41.2 | 41.2 | AMP-binding protein | *Paenibacillus anaericanus* | Soil, earthworm gut bacterium | Bacteria | Firmicutes |
| Gene 234* Gene 279** | 72.222 | 4.8 | 4.7 | 36.6 | 36.6 | ATPase, partial | *Bifidobacterium tissieri* | Rousettus aegyptiacus fecal samples | Bacteria | Actinobacteria |
| Gene 235* Gene 235** | 28.788 | 0.49 | 1.18E-18 | 40.4 | 92.4 | Hypothetical protein C0033 17500 | *Clostridium sp.* chh4-2 | Isolated from gut of a healthy Taiwan adult | Bacteria | Firmicutes |
| Gene 256* | 30.159 | NA | 0.22 | NA | 40.8 | 2OG-Fe(II) oxygenase | *Verrucomicrobia bacterium* | Angelo Coast Range Reserve, CA: meadow soil | Bacteria | Verrucomicrobia |
| Gene 263* | 63.844 | NA | 3.08E-144 | NA | 425 | Hypothetical protein EKK58 08320 | *Ca’* Dependentiae bacterium | Secondary wastewater | Bacteria | Unclassified |
| Gene 275* | 71.875 | NA | 3.79E-24 | NA | 96.7 | Hypothetical protein CBD58 00585 | *Alphaproteobacteria bacterium* TMED198 | Mediterranean Sea | Bacteria | Proteobacteria |
| Gene 36** | 49.135 | NA | 6.79E-83 | NA | 262 | Hypothetical protein CBC22 04800 | *Alphaproteobacteria bacterium* TMED62 | Mediterranean Sea | Bacteria | Proteobacteria |
| Gene 37** | 48.649 | NA | 5.02E-98 | NA | 304 | Hypothetical protein CBC22 04805 | *Alphaproteobacteria bacterium* TMED62 | Mediterranean Sea | Bacteria | Proteobacteria |
| Gene 44** | 32.903 | NA | 4.37E-41 | NA | 155 | Hypothetical protein CBC22 04835 | *Alphaproteobacteria bacterium* TMED62 | Mediterranean Sea | Bacteria | Proteobacteria |
| Gene 64** | 31.933 | NA | 1.05E-04 | NA | 57 | Vomp family autotransporter, partial | *Bartonella birtlesii* | Human pathogen | Bacteria | Proteobacteria |
| Gene 72** | 28.025 | NA | 7.67E-13 | NA | 75.5 | 2OG-Fe(II) oxygenase | *Ca’* Rokubacteria bacterium RIFCSPLOWO2 12 FULL 69 21 | Subsurface microbe (low Oxygen environment) | Bacteria | Candidatus Rokubacteria |
| Gene 83** | 38.017 | NA | 1.94E-09 | NA | 66.2 | MFS transporter | *Bacterium BRH c32* | Opalinus Clay rock porewater BRC-3 borehole | Bacteria | Unclassified |
| Gene 118** | 41.86 | NA | 1.43E-28 | NA | 111 | Ribonuclease HI family protein | *Actinobacteria bacterium* | Temperate grassland biome | Bacteria | Actinobacteria |
| Gene 158** | 36.962 | NA | 6.28E-95 | NA | 350 | DUF5011 domain-containing protein | Endosymbiont of Ridgeia piscesae | Hydrothermal vent | Bacteria | Proteobacteria |
| Gene 159** | 35.385 | NA | 9.57E-20 | NA | 101 | Leucine-rich repeat domain-containing protein | *Brachyspira hampsonii* | Pathogens of animals | Bacteria | Spirochaetes |
| Gene 197** | 38.462 | NA | 0.19 | NA | 40.4 | Hypothetical protein DCF16 18155 | *Alphaproteobacteria bacterium* | Microbial mat from Antartica | Bacteria | Proteobacteria |
| Gene 215** | 33.929 | NA | 5.74E-20 | NA | 92 | Hypothetical protein A7982 11841 | *Minicystis rosea* | Soil | Bacteria | Proteobacteria |
| Gene 223** | 55.153 | NA | 1.79E-137 | NA | 405 | Guanosine monophosphate reductase | *Ca’* Aerophobetes bacterium | Marine subsurface aquifer (North Pond) | Bacteria | Candidatus |
| Gene 256** | 48.571 | NA | 3.5 | NA | 35.4 | Hypothetical protein | *Flavobacteriaceae bacterium* | Unknown | Bacteria | Bacteroidetes |
| Gene 260** | 44.444 | NA | 2.02E-05 | NA | 53.9 | Peptidylprolyl isomerase | *Campylobacter concisus* | Oral flora of Humans, Anaerobic or microaerobic | Bacteria | Proteobacteria |
| Gene 278** | 28.788 | NA | 0.48 | NA | 40.4 | Hypothetical protein C0033 17500 | *Clostridium sp.* chh4-2 | Human Gut | Bacteria | Firmicutes |
| Gene 287** | 33.333 | NA | 1.17E-07 | NA | 58.5 | Two pore domain potassium channel family protein | *Roseomonas sp.* HF4 | Sediment | Bacteria | Other Bacteria |
| Gene 296** | 32.927 | NA | 6.5 | NA | 37.4 | Helix-turn-helix transcriptional regulator | *Chryseobacterium sp.* IHB B 17019 | Isolated from gut of a healthy human | Bacteria | Bacteroidetes |
| Gene 299** | 37.662 | NA | 0.54 | NA | 41.6 | MULTISPECIES: glycoside hydrolase family 32 protein | *Unclassified Streptococcus* | Unknown | Bacteria | Firmicutes |

* vSAG1.JdFR

** vSAG8.JdFR

Detailed protein BLAST results are enlisted in vSAG1.JdFR annotation and vSAG8.JdFR annotation excel sheets.
