# Supplementary material for: Genomics discovery of giant fungal viruses from subsurface oceanic crustal fluids
Source: ISME Commun. 2023 Feb 3;3:10. doi: 10.1038/s43705-022-00210-8 (PMC9894930; doi:10.1038/s43705-022-00210-8)
Supplement: Supplementary file 9 — Table S8 [file 43705_2022_210_MOESM9_ESM.docx]

Table S8: List of genes matching Eukaryote: vSAG1.JdFR and vSAG8.JdFR

| **GeneID** | **%ID** | **E-Value [vSAG1.JDFR]** | **E-Value [vSAG8.JDFR]** | **Bitscore [vSAG1.JDFR]** | **Bitscore [vSAG8.JDFR]** | **Annotation** | **Phyletic Affiliations** | **Environment** | **Kingdom** | **Phylum** |
| --- | --- | --- | --- | --- | --- | --- | --- | --- | --- | --- |
| Gene 3* Gene 2** | 29.63 | 0.026 | 0.025 | 47.8 | 47.8 | WD40 repeat-containing protein | Tieghemostelium lacteum | Unknown | Eukaryota | Dictyosteliales |
| Gene 7* Gene 6** | 31.304 | 1.52E-12 | 1.50E-12 | 74.7 | 74.7 | Hypothetical protein THAOC 02501 | Thalassiosira oceanica | Marine | Eukaryota | Bacillariophyta |
| Gene 28* Gene 27** | 32.065 | 1.92E-16 | 1.89E-16 | 85.5 | 85.5 | Secreted nuclease | Trichomonas vaginalis G3 | Human Pathogen | Eukaryota | Trichomonadida |
| Gene 51* Gene 112** | 35.065 | 2.3 | 2.3 | 38.5 | 38.5 | Uncharacterized protein LOC106424536 | Brassica napus | Terrestrial Plant | Eukaryota | Streptophyta |
| Gene 95* Gene 297** | 37.5 | 2.28E-50 | 2.26E-50 | 182 | 182 | Hypothetical protein PPERSA 11147 | Pseudocohnilembus persalinus | Marine | Eukaryota | Oligohymenophorea |
| Gene 123* Gene 198** | 27.869 | 3.19E-07 | 3.17E-07 | 60.5 | 60.5 | Peroxisome bioproteinsis factor 10, variant 2 | Puccinia graminis f. sp. tritici | Plant pathogen | Eukaryota | Basidiomycota |
| Gene 174* Gene 41** | 44.643 | 7.52E-06 | 7.47E-06 | 55.5 | 55.5 | E3 ubiquitin-protein ligase rnf8-A-like | Agrilus planipennis | Terrestrial insect | Eukaryota | Arthropoda |
| Gene 188* Gene 55** | 29.577 | 7.2 | 7.1 | 36.2 | 36.2 | Hypothetical protein LY89DRAFT 109012 | Phialocephala scopiformis | Terrestrial Fungi | Eukaryota | **Ascomycota** |
| Gene 190* Gene 57** | 32.143 | 7.4 | 7.3 | 37.7 | 37.7 | Ubiquitin-protein ligase E3 | Schizosaccharomyces cryophilus OY26 | Isolate was a contaminant | Eukaryota | **Ascomycota** |
| Gene 195* Gene 62** | 30.233 | 0.55 | 0.55 | 42.7 | 42.7 | Regulatory protein MIG1 | Cryptococcus wingfieldii CBS 7118 | Terrestrial Fungi | Eukaryota | Basidiomycota |
| Gene 211* Gene 248** | 43.396 | 0.62 | 1.13E-07 | 39.3 | 60.1 | Hypothetical protein FOG51 01048 | Hanseniaspora uvarum | Terrestrial Plant | Eukaryota | **Ascomycota** |
| Gene 212* | 40 | 1.15E-07 | NA | 60.1 | NA | Unnamed protein product | Vitrella brassicaformis CCMP3155 | Marine-Great Barrier Reef | Eukaryota | Chromerida |
| Gene 215* | 44.828 | 2.48E-05 | NA | 54.3 | NA | Poly [ADP-ribose] polymerase 11-like | Pomacea canaliculata | Freshwater | Eukaryota | Mollusca |
| Gene 218* Gene 160** | 47.727 | 3.01E-07 | 2.97E-07 | 57 | 57 | RING/U-box superfamily protein | Arabidopsis thaliana | Terrestrial Plant | Eukaryota | Streptophyta |
| Gene 240* | 29.71 | 8.21E-10 | NA | 67.4 | NA | Unnamed protein product | Brassica rapa | Terrestrial Plant | Eukaryota | Streptophyta |
| Gene 260* | 65.714 | 8.27E-04 | NA | 44.3 | NA | Microtubule-associated protein 4 | Liparis tanakae | Temperate and cold water | Eukaryota | Chordata |
| Gene 268* | 35.052 | 1.8 | NA | 39.7 | NA | Dolichyl-phosphate-mannose-protein mannosyltransferase-domain-containing protein | Catenaria anguillulae PL171 | Nematode parasite | Eukaryota | Blastocladiomycota |
| Gene 229** | 41.463 | NA | 6.7 | NA | 34.7 | Unnamed protein product | Trichobilharzia regenti | Pathogen of birds and Human | Eukaryota | Platyhelminthes |
| Gene 239** | 41.401 | NA | 0 | NA | 754 | Unnamed protein product | Triticum turgidum subsp. durum | Terrestrial Plant | Eukaryota | Streptophyta |
| Gene 239** | 40.457 | NA | 0 | NA | 741 | RNA polymerase II largest subunit | Cavenderia fasciculata | Soil | Eukaryota | Acytosteliales |
| Gene 247** | 44.681 | NA | 0.74 | NA | 38.9 | Oleate-activated transcription factor 1 | Hanseniaspora uvarum | Terrestrial Plant | Eukaryota | **Ascomycota** |
| Gene 254** | 28.571 | NA | 6.2 | NA | 39.7 | Histone deacetylase 19 | Glycine max | Terrestrial Plant | Eukaryota | Streptophyta |
| Gene 258** | 37.037 | NA | 0.032 | NA | 42.7 | Peroxidase 73 | Morella rubra | Terrestrial Plant | Eukaryota | Streptophyta |
| Gene 261** | 40.816 | NA | 1.25E-58 | NA | 206 | Predicted protein | Naegleria gruberi strain NEG-M | Freshwater | Eukaryota | Heterolobosea |
| Gene 262** | 37.586 | NA | 1.32E-65 | NA | 228 | Iron-sulfur clusters transporter atm1, mitochondrial | Exophiala oligosperma | Human Pathogen | Eukaryota | **Ascomycota** |

* vSAG1.JdFR

** vSAG8.JdFR

Detailed protein BLAST results are enlisted in vSAG1.JdFR annotation and vSAG8.JdFR annotation excel sheets.
