# Supplementary material for: Genomics discovery of giant fungal viruses from subsurface oceanic crustal fluids
Source: ISME Commun. 2023 Feb 3;3:10. doi: 10.1038/s43705-022-00210-8 (PMC9894930; doi:10.1038/s43705-022-00210-8)
Supplement: Supplementary file 10 — Table S9 [file 43705_2022_210_MOESM10_ESM.docx]

Table S9: List of vSAG1.JdFR and vSAG8.JdFR genes acquired from eukaryotes via horizontal gene transfer.

| **GeneID** | **%ID** | **E-value** | **Bitscore** | **Query Length** | **Subject Length** | **Annotation** | **Kingdom**  **(phylum)** | **Order** |
| --- | --- | --- | --- | --- | --- | --- | --- | --- |
| Gene_173*/ Gene_40**^@^ | 64.43 | 1.04E-64 | 206 | 206 | 151 | Ubiquitin-conjugating_enzyme_E2-24_kDa-like_isoform_X2_[Mucor ambiguus] | Eukaryota  (Acomycota) | Schizosaccharomycetes |
| Gene_262**^^^ | 37.586 | 7.98E-66 | 228 | 305 | 725 | Iron-sulfur clusters transporter atm1,_mitochondrial  [Exophiala_oligosperma] | Eukaryota  (Ascomycota) | Chaetothyriales |
| Gene_123^*^/ Gene_198**^!^ | 27.869 | 3.19E-07 | 60.5 | 163 | 178 | Peroxisome_bioproteinsis_factor_10,_variant_2_  [Puccinia_graminis_f._sp._tritici] | Eukaryota  (Basidiomycota) | Pucciniomycetes |
| Gene_195^*^/ Gene_62**^#^ | 30.233 | 0.55 | 42.7 | 117 | 121 | Regulatory_protein_MIG1_  [Cryptococcus_wingfieldii_CBS_7118] | Eukaryota  (Basidiomycota) | Tremellomycetes |

* vSAG1.JdFR; **vSAG8.JdFR

^^^ Figure 3 B(i)

^!^ Figure 3 B(ii)

^@^ Figure 3 B(iii)

^#^ Figure 3 B(iv)
