# Supplementary material for: Genomics discovery of giant fungal viruses from subsurface oceanic crustal fluids
Source: ISME Commun. 2023 Feb 3;3:10. doi: 10.1038/s43705-022-00210-8 (PMC9894930; doi:10.1038/s43705-022-00210-8)
Supplement: Supplementary file 11 — Table S10 [file 43705_2022_210_MOESM11_ESM.docx]

Table S10: 28S rRNA gene sequences recovered from two assembled metagenome from IODP boreholes U1368A and U1362B at the Juan de Fuca Ridge.

| **GeneID** | **Length (bps)** | **Identity (Silva)** | **Taxonomy** | **Copy number (JGI)** | |
| --- | --- | --- | --- | --- | --- |
| **Partial 28S rRNA (U1368A)** | | | | | |
| JGI24020J35080_1059404 | 457 | NA^*^ | *Could not be classified* | | 16 |
| JGI24020J35080_1101045 | 284 | NA^*^ | *Could not be classified* | | 8 |
| **Partial 28S rRNA (U1368B)** | | | | | |
| JGI24019J35510_1012583 | 1660 | 50.03% | *Unclassified* | | 88 |
| JGI24019J35510_1020216 | 1085 | 37.29% | *Unclassified* | | 71 |
| JGI24019J35510_1040447 | 626 | 41.89% | *Unclassified* | | 116 |
| JGI24019J35510_1048180 | 448 | 33.17% | *Unclassified* | | 78 |
| JGI24019J35510_1006172 | 443 | 30.04% | *Eukaryota;Fungi;Dikarya;****Ascomycota****;Pezizomycotina* | | 28 |
| JGI24019J35510_1099350 | 355 | 41.41% | *Unclassified* | | 8 |
| JGI24019J35510_1108724 | 339 | 41.46% | *Unclassified* | | 10 |
| JGI24019J35510_1122927 | 339 | 40.83% | *Unclassified* | | 22 |
| JGI24019J35510_1156810 | 318 | 37.19% | *Unclassified* | | 6 |
| JGI24019J35510_1171710 | 275 | 45.45% | *Unclassified* | | 10 |
| JGI24019J35510_1181864 | 268 | 42.19% | *Unclassified;* | | 18 |
| JGI24019J35510_1012447 | 218 | 99.74 | *Eukaryota;Amorphea;Obazoa;Opisthokonta;Nucletmycea;Fungi;Dikarya;****Ascomycota****;Pezizomycotina;Sordariomycetes;Hypocreales;Cordycipitaceae* | | 47 |
| JGI24019J35510_1007819 | 202 | 99.74% | *Eukaryota;Fungi;Dikarya;****Ascomycota****;Pezizomycotina;Eurotiomycetes;Eurotiomycetidae;Eurotiales;Aspergillaceae;Aspergillus* | | 87 |
| JGI24019J35510_1021349 | 202 | 99.50% | *Eukaryota;Amorphea;Obazoa;Opisthokonta;Nucletmycea;Fungi;Dikarya;****Ascomycota****;Pezizomycotina;Eurotiomycetes;Eurotiales;Aspergillaceae;Aspergillus* | | 44 |

* 28S RNA gene sequences submitted to Silva ACT: Alignment, Classification and Tree Service server could not perform analysis.

The sequences were also submitted to NCBI, BLASTn (database: rRNA/ITS) analysis but no matches were found for them.
