# Supplementary material for: Genomics discovery of giant fungal viruses from subsurface oceanic crustal fluids
Source: ISME Commun. 2023 Feb 3;3:10. doi: 10.1038/s43705-022-00210-8 (PMC9894930; doi:10.1038/s43705-022-00210-8)
Supplement: Supplementary file 12 — Table S11 [file 43705_2022_210_MOESM12_ESM.docx]

Table S11: Internal Transcribe Spacer (ITS) sequences recovered from the assembled metagenome from IODP borehole U1362B at the Juan de Fuca Ridge.

| **Scaffold ID** | **Length (bps)** | **Identity (Silva)** | **Taxonomy** | **Copy number (JGI)** |
| --- | --- | --- | --- | --- |
| JGI24019J35510_1007819 | 533 | 90.60% | *Eukaryota; Fungi; Dikarya;* ***Ascomycota****; Pezizomycotina;*  *Eurotiomycetes; Eurotiomycetidae; Eurotiales; Aspergillaceae* | 86 |
| JGI24019J35510_1012447 | 447 | 97.10%% | *Eukaryota; Fungi; Dikarya;* ***Ascomycota****; Pezizomycotina; Sordariomycetes; Hypocreomycetidae; Hypocreales; Cordycipitaceae* | 47 |
| JGI24019J35510_1021349 | 502 | 94.40% | *Eukaryota; Fungi; Dikarya;* ***Ascomycota****; Pezizomycotina; Eurotiomycetes; Eurotiomycetidae; Eurotiales; Aspergillaceae* | 44 |
