# Supplementary material for: Genomics discovery of giant fungal viruses from subsurface oceanic crustal fluids
Source: ISME Commun. 2023 Feb 3;3:10. doi: 10.1038/s43705-022-00210-8 (PMC9894930; doi:10.1038/s43705-022-00210-8)
Supplement: Supplementary file 13 — Table S12 [file 43705_2022_210_MOESM13_ESM.docx]

Table S12: Concentration of major and minor elements in fluid samples collected from the Hole U1362B CORK on the Juan de Fuca Ridge flank in comparison to bottom seawater**.** Chemical comparison of Hole U1362B CORK fluids collected via syringe sampler from fluids freely venting from the top of the open CORK (this study) and via *in situ* pumping/filtration of fluids through the Tefzel umbilical (sample SSF18 in Nigro *et al.*^1^ and Wheat *et al.*^2^) to background bottom seawater (BSW) at this location (shows that the two methods of collecting crustal fluids resulted in nearly identical sample quality. Data in this study provided courtesy of Geoff Wheat, University of Alaska Fairbanks

|  | **This study** | **Jungbluth**^3^**/Nigro**^1^ | **BSW** |
| --- | --- | --- | --- |
| Year collected | 2011 | 2011 | 2010 |
| ROV Jason dive # | J2-569 | J2-571 | n.a. |
| Collection method | Syringe | In situ filtration | n.a. |
| Fluid source depths (mbsf^a^) | 279-359 | 279-359 | n.a. |
| Na (mM) | 452 | 462 | 467 |
| Ca (mM) | 54.3 | 55.4 | 10.3 |
| Mg (mM) | 2.4 | 2.5 | 52.6 |
| SO4 (mM) | 18.6 | 18.6 | 28.1 |
| Si (µM) | 1218 | 1144 | 190 |
| Fe (µM) | 1.4 | 1.3 | 0 |

^a^ mbsf, meters below seafloor
