# Supplementary figures and images for: Genomics discovery of giant fungal viruses from subsurface oceanic crustal fluids
Source: ISME Commun. 2023 Feb 3;3:10. doi: 10.1038/s43705-022-00210-8 (PMC9894930; doi:10.1038/s43705-022-00210-8)

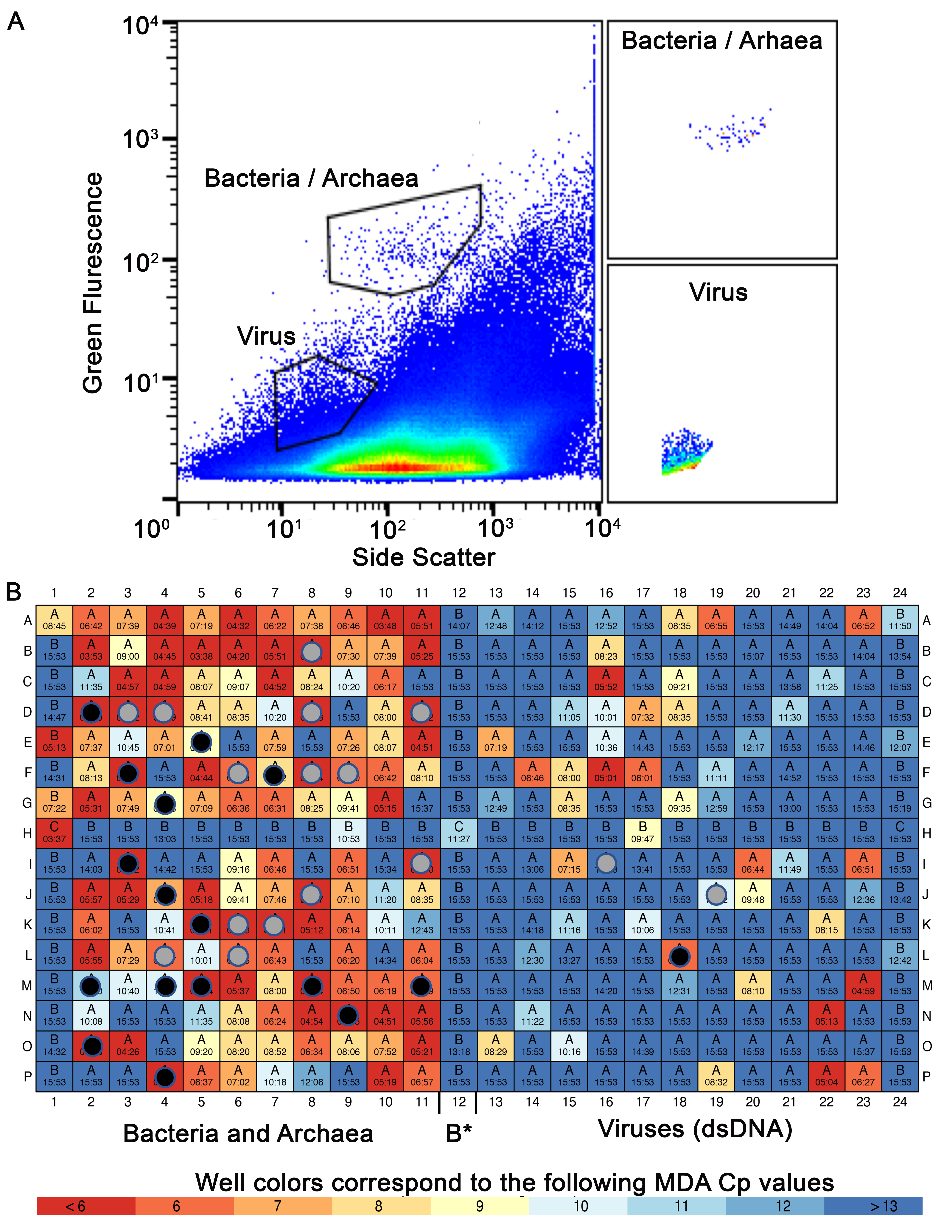

Supplement: Supplementary file 14 — Figure S1 [file 43705_2022_210_MOESM14_ESM.jpg]

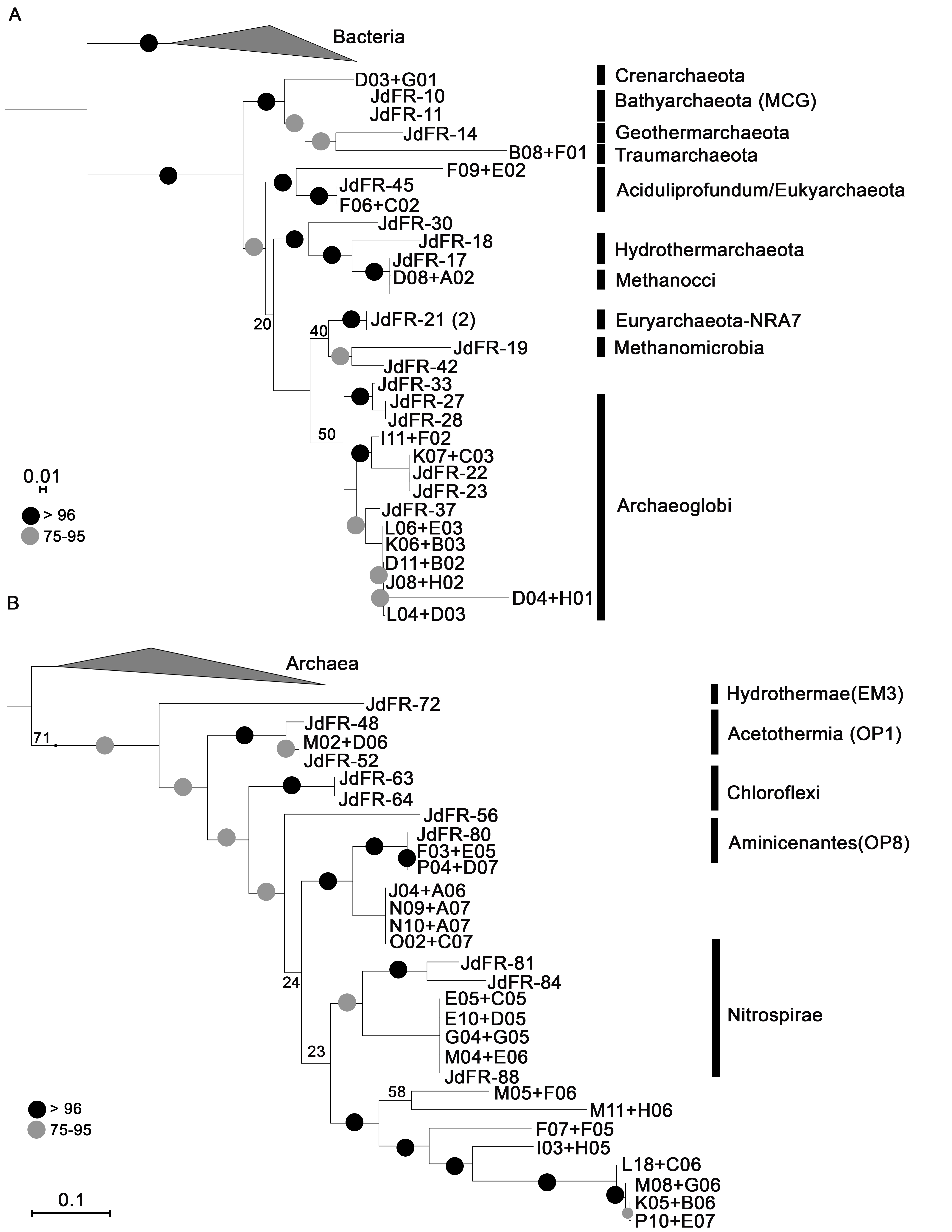

Supplement: Supplementary file 15 — Figure S2 [file 43705_2022_210_MOESM15_ESM.jpg]

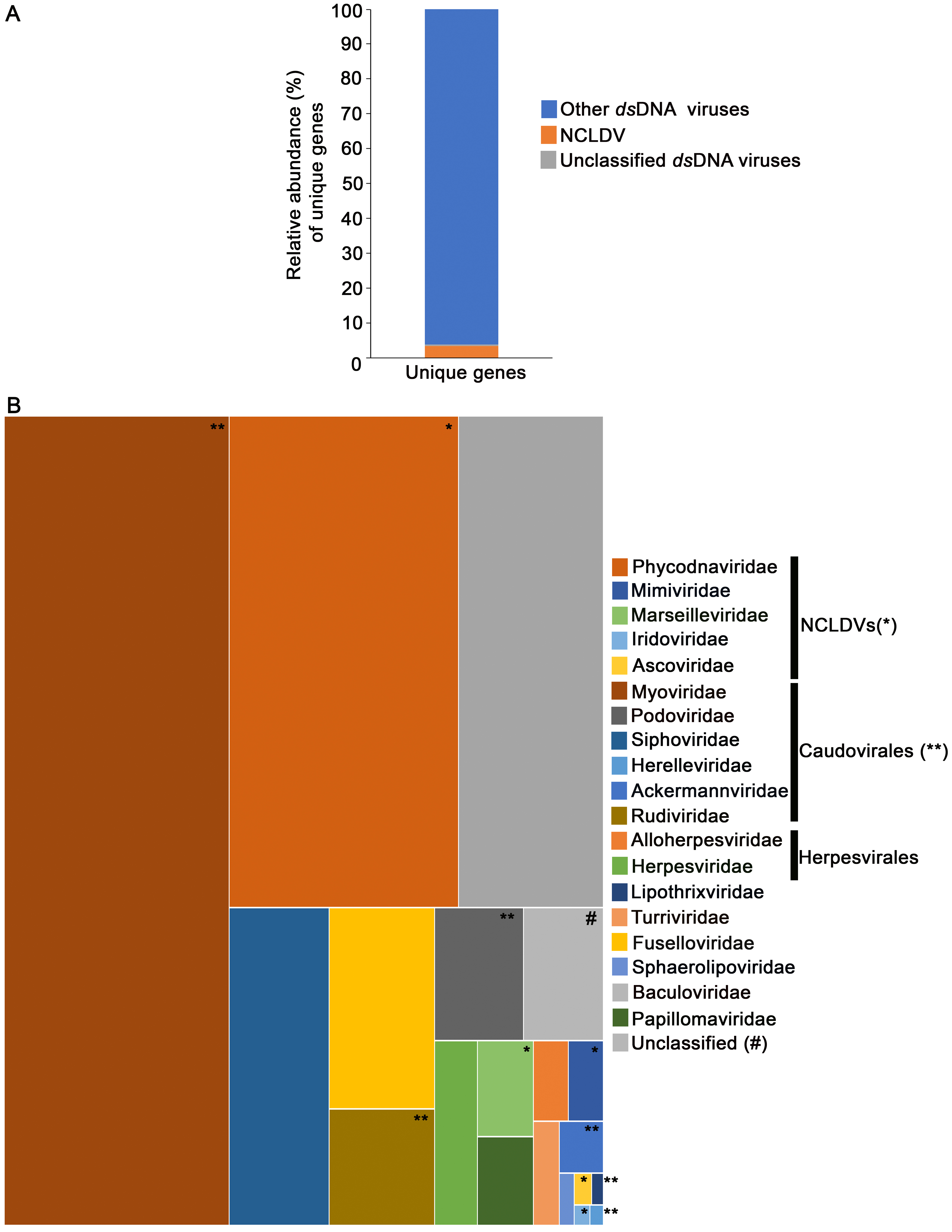

Supplement: Supplementary file 16 — Figure S3 [file 43705_2022_210_MOESM16_ESM.jpg]

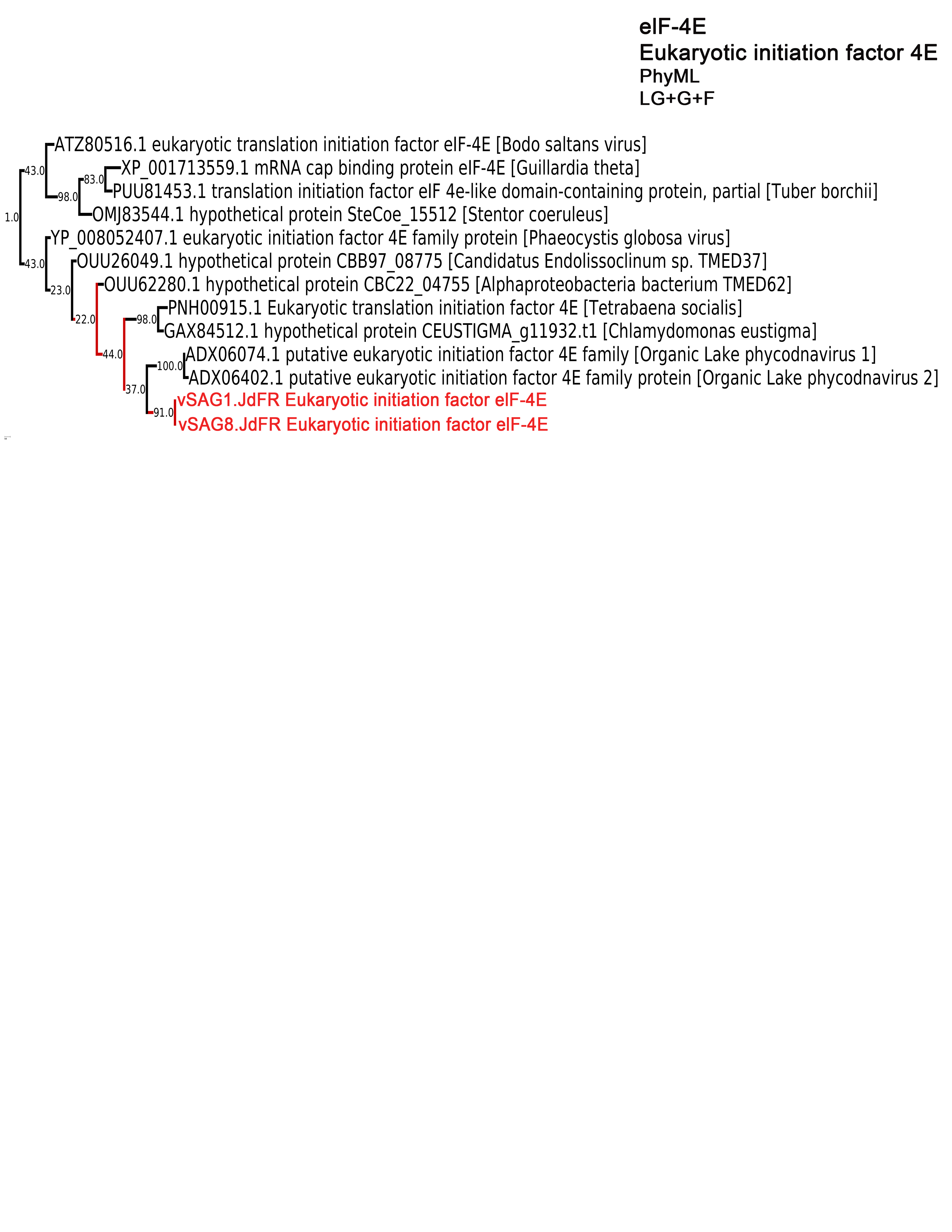

Supplement: Supplementary file 17 — Figure S4 [file 43705_2022_210_MOESM17_ESM.jpg]

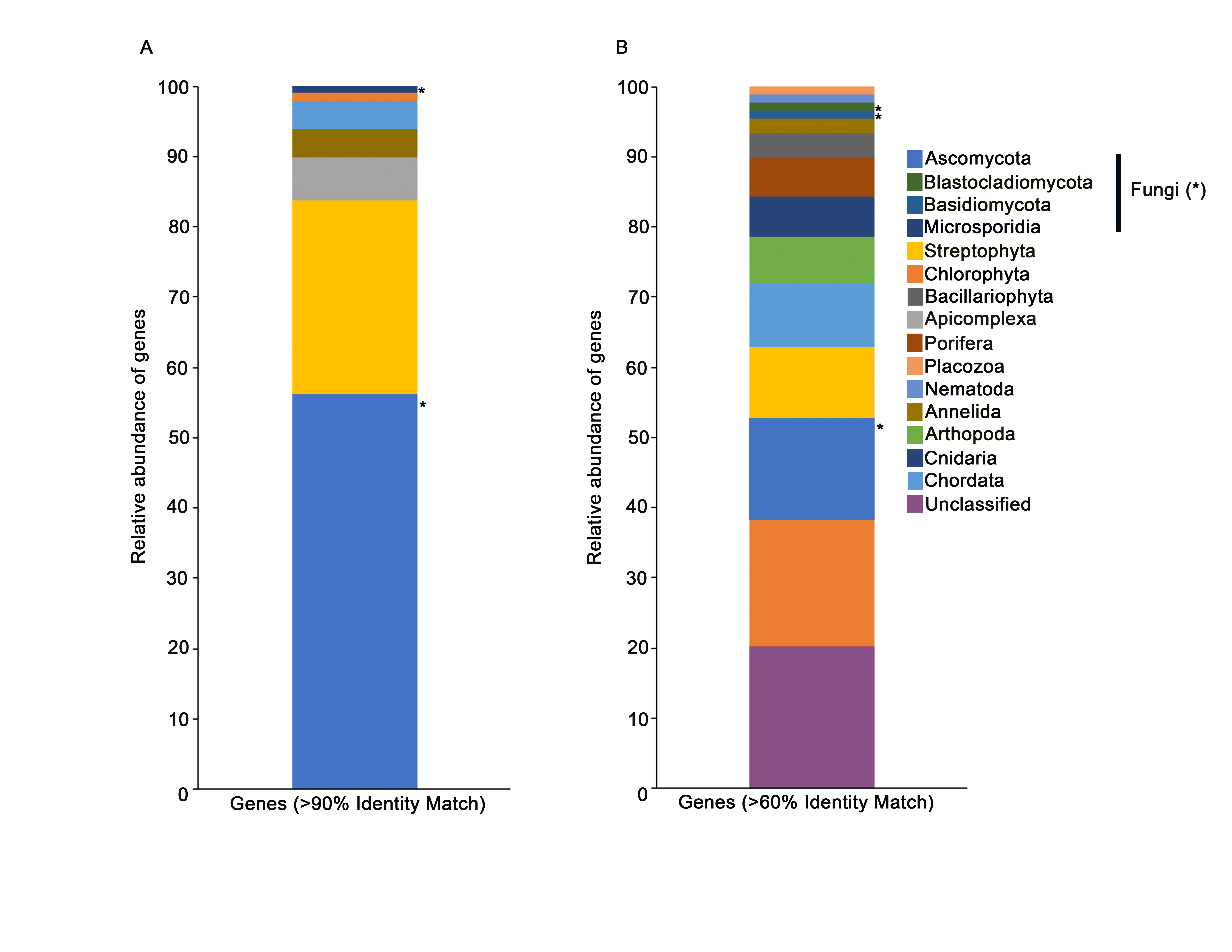

Supplement: Supplementary file 18 — Figure S5 [file 43705_2022_210_MOESM18_ESM.png]
